# Identifying a gene expression signature of frequent COPD exacerbations in peripheral blood using network methods

**DOI:** 10.1186/s12920-014-0072-y

**Published:** 2015-01-13

**Authors:** Jarrett D Morrow, Weiliang Qiu, Divya Chhabra, Stephen I Rennard, Paula Belloni, Anton Belousov, Sreekumar G Pillai, Craig P Hersh

**Affiliations:** Channing Division of Network Medicine, Brigham and Women’s Hospital, 181 Longwood Avenue, Boston, MA 02115 USA; Division of Biomedical Informatics, University of California, San Diego, CA USA; University of Nebraska Medical Center, Omaha, NE USA; Genentech, Member of the Roche Group, South San Francisco, CA USA; Roche Innovation Center, Penzberg, Germany; Hoffman La Roche, Nutley, NJ USA; Current address: Eli Lilly and Company, Indianapolis, IN USA

**Keywords:** Network analysis, Chronic obstructive pulmonary disease, Gene expression profiling, Biomarker

## Abstract

**Background:**

Exacerbations of chronic obstructive pulmonary disease (COPD), characterized by acute deterioration in symptoms, may be due to bacterial or viral infections, environmental exposures, or unknown factors. Exacerbation frequency may be a stable trait in COPD patients, which could imply genetic susceptibility. Observing the genes, networks, and pathways that are up- and down-regulated in COPD patients with differing susceptibility to exacerbations will help to elucidate the molecular signature and pathogenesis of COPD exacerbations.

**Methods:**

Gene expression array and plasma biomarker data were obtained using whole-blood samples from subjects enrolled in the Treatment of Emphysema With a Gamma-Selective Retinoid Agonist (TESRA) study. Linear regression, weighted gene co-expression network analysis (WGCNA), and pathway analysis were used to identify signatures and network sub-modules associated with the number of exacerbations within the previous year; other COPD-related phenotypes were also investigated.

**Results:**

Individual genes were not found to be significantly associated with the number of exacerbations. However using network methods, a statistically significant gene module was identified, along with other modules showing moderate association. A diverse signature was observed across these modules using pathway analysis, marked by differences in B cell and NK cell activity, as well as cellular markers of viral infection. Within two modules, gene set enrichment analysis recapitulated the molecular signatures of two gene expression experiments; one involving sputum from asthma exacerbations and another involving viral lung infections. The plasma biomarker myeloperoxidase (MPO) was associated with the number of recent exacerbations.

**Conclusion:**

A distinct signature of COPD exacerbations may be observed in peripheral blood months following the acute illness. While not predictive in this cross-sectional analysis, these results will be useful in uncovering the molecular pathogenesis of COPD exacerbations.

**Electronic supplementary material:**

The online version of this article (doi:10.1186/s12920-014-0072-y) contains supplementary material, which is available to authorized users.

## Background

Chronic obstructive pulmonary disease (COPD) is characterized by progressive airflow obstruction accompanied by chronic inflammation. It is one of the leading causes of morbidity and mortality worldwide and is often caused by environmental exposure such as cigarette smoke [1]. COPD exacerbations, periods of acute deterioration, are a major reason for COPD mortality and a major source of the high healthcare expenditure in patients with COPD. Acute exacerbations of COPD are characterized by symptoms of shortness of breath, cough, and sputum production. Although these exacerbations are often caused by bacterial or viral infections [2] or inhaled particles, the variability in occurrence within COPD patients and the familial aggregation of exacerbations indicate that other factors such as genetics are important in determining the onset, severity and frequency [3]. Also, the frequency of acute exacerbations appears to be a stable trait [4], supporting genetic susceptibility, and loci associated with COPD exacerbations have been identified [5,6].

Despite the fact that gene expression data from lung tissues should provide greater sensitivity to detect the molecular signature of COPD exacerbations, COPD is a systemic disease, and blood is more accessible for genomics and biomarkers studies in large scale clinical trials and potentially in clinical practice than is lung tissue samples. Previous attempts to study lung disease via whole blood experiments have been successful while studying asthma [7] and idiopathic pulmonary fibrosis [8,9]. Further supporting the use of blood expression profiling, previous COPD studies have documented differential expression in overlapping genes from both blood and lung samples [10,11]. Gene expression in peripheral blood has been associated with COPD and related phenotypes [12].

Network medicine approaches provide a roadmap towards the understanding of complex diseases by studying interacting gene sets and pathways, instead of individual genetic determinants [13,14]. Network medicine techniques have been applied to the study of COPD [15].

We hypothesized that we could identify a signature of frequent COPD exacerbations using gene expression data and protein biomarker data, both collected from peripheral blood samples. The goal is to use network methods to understand the molecular pathogenesis of COPD exacerbations, and perhaps predict onset through minimally invasive means. This study built upon the prior publications involving the use of peripheral blood to examine the molecular pathogenesis of COPD and other complex diseases [8,11,12], and leveraged the power of network analysis methods to uncover gene expression signatures.

## Methods

### Study population

This analysis used expression data from 248 Caucasian COPD subjects from the Treatment of Emphysema with a Selective Retinoid Agonist (TESRA), a randomized controlled trial of palovarotene for treatment of COPD (clinicaltrials.gov identifier NCT00413205) [16,17]. TESRA subjects were former smokers with COPD who experienced two or fewer exacerbations requiring outpatient treatment with antibiotics or oral steroids or one exacerbation requiring hospitalization within the prior year. The total number of subjects in the study was 410. In our analysis, the number of exacerbations in the year prior to enrollment was considered both as a linear variable (0,1,2) and as a binary variable (0 vs. 1 or more). The baseline blood samples, from which our analysis data were obtained, were collected prior to randomization and any treatment associated with the clinical trial. Quantitative emphysema measurements were performed on chest computed tomography (CT) scans, including percent of lung with attenuation (%LAA) ≤ −910 HU and mean lung attenuation at the 15th percentile on lung attenuation curve (Perc15). The baseline phenotype data in our analyses were obtained prior to randomization and any treatment. All subjects provided written informed consent. TESRA was approved by the Institutional Review Boards at all participating centers (see Additional file 1).

### Gene expression data

RNA was isolated from whole blood samples collected in PaxGene RNA tubes (PreAnalytiX, Franklin Lakes, NJ). Gene expression data were obtained using the Affymetrix Human Genome U133 Plus 2.0 Array. Data were available for 309 samples, including 39 replicates, as outlined in Additional file 2: Table S1. Of the 270 remaining unique arrays, three were found to be outliers during the quality control process and were discarded. All 54,675 probes passing quality control were retained regardless of level of variance across samples. These data were background corrected and quantile normalized using the robust multi-array average (RMA) method via the R Bioconductor package affy [18]. The 249 subjects that self-identified as Caucasian were included. Using information from a GWAS, one additional subject was omitted based on a genetic ancestry that did not cluster with the other Caucasian subjects, leaving 248 subjects for analysis.

### Protein biomarkers

A total of 140 plasma biomarkers were measured in duplicate at Rules Based Medicine (Austin, TX) and Quest Diagnostics (Valencia, CA), as previous described [17]. Biomarker data are available for 245 of the 248 subjects with associated gene expression data. Any biomarker with greater than 10% and less than 95% of its values below the lower limit of quantitation (LLOQ) were converted to a binary present vs. absent variable (n = 25). Two biomarkers with greater than 95% values below the LLOQ were excluded from the analysis. An empirical normal quantile transformation was performed on the 113 remaining continuous biomarker values. Model #1 from Additional file 2: Table S2 was used to assess the association of biomarker levels with the number of recent exacerbations. Logistic regression was used for the binary biomarker values, and linear regression was used for the continuous biomarker values.

### Gene expression analysis

Microarray batch effects visible from MDS plots were addressed by adjusting for the microarray lot number as a covariate. For each expression probe, biomarker or module eigengene, we fitted a linear regression model to detect association with variables of interest using R statistical software (v 3.0.2). In the microarray data analysis, an empirical Bayes shrinkage method was used to obtain a moderated t-test statistic and its p-value in the R Bioconductor package limma [19]. Adjustment for multiple testing controlled for false discovery rate (FDR) [20]. The regression models for each COPD phenotype are provided in Additional file 2: Table S2.

### Weighted Gene Co-expression Analysis (WGCNA)

The WGCNA method was used to identify groups of probes that have similar expression in the sample population, using the R package WGCNA [21]. In order to identify possible sample outliers, a sample network based on squared Euclidean distances was built. An iterative approach was taken, where samples having a standardized connectivity below −5 were removed [21]. One sample outlier was excluded using this process, leaving 247 subjects. Signed networks were built using biweight midcorrelation as the correlation function, and a soft thresholding power of 12. WGCNA produces a set of modules (labeled by color), each containing a set of unique genes. The module eigengene is defined as the first principal component of the expression matrix of the probes within the module. For association tests, the module eigengenes are included in the regression models from Additional file 2: Table S2, similar to the expression probes, and adjustment for multiple testing was performed using Bonferroni correction, where the burden is reduced in comparison with a probe-based analysis. Driver or hub genes within each module are the probes with both high gene significance (GS) and high module membership (MM) metrics, represented by the p-value for the test of gene expression association with phenotype, and the correlation of gene expression with the module eigengene, respectively.

### Pathway analysis

We examined enrichment of curated gene sets from the gene ontology (GO) and Kyoto Encyclopedia of Genes and Genomes (KEGG) databases in the gene groups from association tests or the co-expression modules using a hypergeometric test in the R Bioconductor package GeneAnswers [22]. In the GO analysis, only the biological process (BP) category was considered. Further examination of the gene sets was performed using the gene set enrichment analysis (GSEA) to examine up- and down-regulated genes common to previous experiments and curated gene sets within all available MSigDB collections [23]. The query gene lists (top association test results or module genes) were preranked according to their log fold change values from the gene expression association tests. GSEA was performed on both a set of all probes and subsets of probes from WGCNA modules, using a similar concept to the GO and KEGG analyses. The Cytoscape plug-in EnrichmentMap [24] was used to plot the relationships between the individual GSEA results, as well as between the sets of results from different analyses.

## Results

### Gene expression association with phenotype variables

Characteristics of study subjects are shown in Table 1. The top five expression probesets ranked by p-value for association with number of exacerbations in the past 12 months are provided in Additional file 2: Table S3. After controlling for FDR, no probes were statistically significant. The results for all other models are also provided in Additional file 2: Table S3, where only one probe across the COPD-related phenotypes was found to be significant at FDR < 0.05, for the six minute walk test. This probe did not have a gene annotation and maps to a region with no obvious biological significance. Enrichment tests were performed using the set of genes within the regression results having a p < 0.05 for the exacerbation phenotype (Table 2). Although the KEGG results demonstrate an enrichment of autoimmune-related genes, the overall results indicate a lack of specificity, as demonstrated by the presumably-unrelated diseases represented. In addition, the GO results are very broad, with processes related to protein ubiquitination and regulation of calcium concentration being dominant. To improve specificity, we used a network approach, whereby genes with similar characteristics may be grouped prior to pathway analysis.

**Table 1:** **TESRA study subjects**

**Demographics**	**n = 248 expression (n = 245/248 biomarker)**	**Association with exacerbations**
Age (years)	66.4 +/− 7.9	linear regression p-value = 0.11
Gender	Female 78	Fisher's Exact Test p-value = 0.86
	Male 170	
Race	Caucasian 100%	NA
Former smokers	100%	NA
Smoking history (pack-years)	47 +/− 24.3	linear regression p-value = 0.02
BMI	26 +/− 4.7	linear regression p-value = 0.39
**COPD outcomes**		
Exacerbations in past 12 months	0 n = 123	
	1 n = 102	
	2 n = 23	
FEV1 (L)	1.34 +/− 0.35	
FEV1 % predicted	48.8 +/− 9.3	
FEV1/FVC	0.43 +/− 0.09	
% LAA ≤ −910 HU	41.65 +/− 16.2	
Perc15 (HU)	−946 +/− 23.6	
TLC (L)	6.9 +/− 1.4	
TLC % predicted	101.6 +/− 15.4	
DLCO (mL/min/mmHg)	12.3 +/− 3.9	
6 minute walk test (m)	317.5 +/− 111.2	
Residual volume % predicted	120.1 +/− 31	
Inspiratory capacity (L)	2.4 +/− 0.67

**Table 2:** **Top categories enriched in association results for exacerbation phenotype (gene n = 999)**

**GO category**	**ID**	**Genes in category**	**p-value**	**FDR q-value**
Protein ubiquitination	GO:0016567	23	2.78E-008	4.42E-006
Purine ribonucleoside triphosphate catabolic process	GO:0009207	17	2.40E-006	4.78E-005
Guanosine-containing compound catabolic process	GO:1901069	16	1.04E-006	2.69E-005
Calcium ion homeostasis	GO:0055074	15	3.16E-007	1.68E-005
Epidermal growth factor receptor signaling pathway	GO:0007173	14	1.18E-007	9.37E-006
Cellular calcium ion homeostasis	GO:0006874	14	1.12E-006	2.69E-005
Activation of protein kinase activity	GO:0032147	10	2.68E-004	2.66E-003
Negative regulation of protein serine/threonine kinase activity	GO:0071901	9	9.97E-007	2.69E-005
**KEGG category**	**ID**	**Genes in category**	**p-value**	**FDR q-value**
Cell adhesion molecules (CAMs)	04514	13	3.86E-003	1.44E-001
B cell receptor signaling pathway	04662	11	2.72E-004	4.75E-002
T cell receptor signaling pathway	04660	11	5.62E-003	1.54E-001
Antigen processing and presentation	04612	10	1.23E-003	5.73E-002
Chagas disease (American trypanosomiasis)	05142	10	1.20E-002	2.23E-001
Primary immunodeficiency	05340	7	5.37E-004	4.75E-002
Allograft rejection	05330	7	7.66E-004	4.75E-002
Autoimmune thyroid disease	05320	7	5.81E-003	1.54E-001

### Network analysis

A weighted gene co-expression network was constructed as outlined in Methods. The final network consisted of 32 modules, ranging in size from 30 to 19,748 probes. Two dendrograms of the module structure are presented in Additional file 2: Figures S1-S2, as the number of probes (54,675) dictated that the network be built in two blocks. There was high correlation within the modules as expected, with the mean module membership (MM), or mean correlation of gene expression with the module eigengene, varying from 0.54 to 0.81 across all modules of interest. The grey module is a grouping of probes with outlying gene expression profiles and was not considered further.

**Figure 1:**
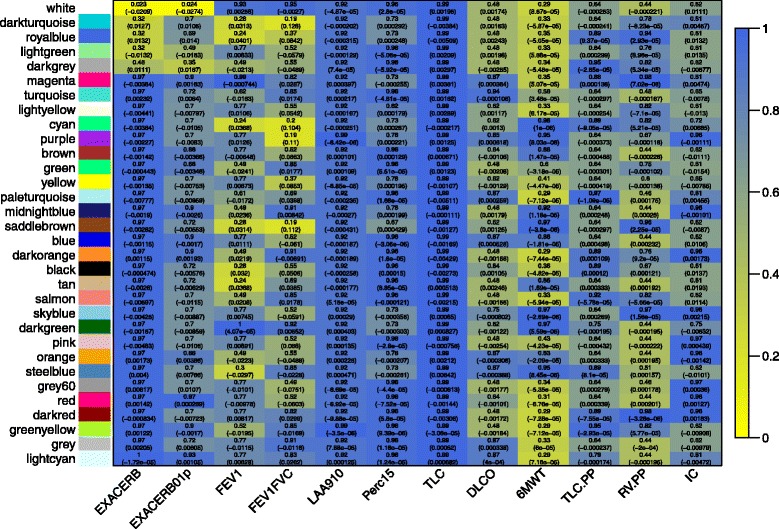
**Heatmap of module association with phenotype variables (color scale for adjusted p-value).** The top number in each cell corresponds to the FDR q-value and the bottom number is the effect from the linear regression. Variable definitions are listed in Additional file 2: Table S2.

Tests of association between COPD phenotypes and the module eigengenes were performed for each model in Additional file 2: Table S2, and summarized in Figure 1. Across all phenotype variables and modules, only the white module (number of probes, n = 57) was significant after controlling for FDR (q-value = 0.02) for the association with the number of exacerbations within the year prior to enrollment. In addition, four other modules were nominally significant (p < 0.05) for the association with exacerbations (royalblue n = 118, lightgreen n = 147, darkturquoise n = 73, darkgrey n = 65) (Additional file 2: Table S4). All probesets in these modules had variance above the lower 5%ile for all probesets. The top modules associated with both exacerbations phenotypes were similar (Additional file 2: Table S5).

Lacking similar support across the results for the other phenotypes, we chose to focus exclusively on the exacerbations phenotype for the remainder of the study. The putative hub genes for the five modules of interest are highlighted in the lists of module genes in Additional file 2: Tables S6-S10. These hub genes with low gene significance (GS) p-values and high module membership (MM) correlations for the five modules include IGHM (immunoglobulin heavy constant mu; GS =0.002, MM = 0.9), KLRD1 (killer cell lectin-like receptor subfamily D, member 1; GS = 0.006, MM = 0.89 and GS = 0.007, MM = 0.88), AFF3 (AF4/FMR2 family, member 3; GS = 0.0014, MM = 0.8), GPR56 (G protein-coupled receptor 56; GS = 0.00024, MM = 0.8), and GBP5 (guanylate binding protein 5; GS = 0.009, MM = 0.77 and GS = 0.016, MM = 0.77) and GBP1 (guanylate binding protein 1, interferon-inducible; GS = 0.028, MM = 0.87).

GO and KEGG pathway analyses were performed on the five modules above. The results are provided in Table 3 for the white module and Additional file 2: Tables S11-S14 for the other four modules. In contrast to the results for the overall gene expression association, the enrichment tests for each module provide more specific functional signatures, with B cell receptor signaling and autoimmune disease being enriched in the results for the white module; B cell pathway is also seen in the lightgreen module results. The darkgrey and darkturquoise module results are highlighted by cytokine activity, with the darkgrey module showing a strong signal for interferon activity and viral response pathways.

**Table 3:** **Top eight pathway analysis results for white module (unique gene symbols n = 32)**

**GO category**	**ID**	**Genes in category**	**p-value**	**FDR q-value**
B cell receptor signaling pathway	GO:0050853	2	0.00072	0.10
Antigen processing and presentation of exogenous peptide antigen via MHC class II	GO:0019886	2	0.0055	0.13
Antigen receptor-mediated signaling pathway	GO:0050851	2	0.011	0.13
Skeletal muscle tissue development	GO:0007519	2	0.029	0.18
Skeletal muscle organ development	GO:0060538	2	0.031	0.18
Clustering of voltage-gated potassium channels	GO:0045163	1	0.0036	0.13
Protein localization to juxtaparanode region of axon	GO:0071205	1	0.0048	0.13
Positive regulation of humoral immune response mediated by circulating immunoglobulin	GO:0002925	1	0.0073	0.13
**KEGG category**	**ID**	**Genes in category**	**p-value**	**FDR q-value**
B cell receptor signaling pathway	04662	4	7.6E-006	0.00018
Cell adhesion molecules (CAMs)	04514	4	7.4E-005	0.00088
Asthma	05310	2	1.4E-003	0.011
Allograft rejection	05330	2	2.1E-003	0.011
Graft-versus-host disease	05332	2	2.5E-003	0.011
Type I diabetes mellitus	04940	2	2.8E-003	0.011
Intestinal immune network for IgA production	04672	2	3.4E-003	0.012
Autoimmune thyroid disease	05320	2	4.0E-003	0.012

**Figure 2:**
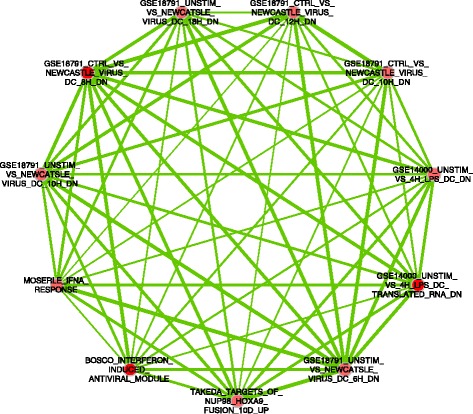
**EnrichmentMap network for GSEA results from darkgrey module.** Nodes correspond to gene sets and edges correspond to an overlap of the genes within two sets.

GSEA was performed on the five modules, and the results meeting the FDR q-value < 0.25 are provided in Table 4. B cell, T-cell and viral response signatures dominate the results, with the white, royalblue and darkgrey modules demonstrating enrichments for viral response gene sets. EnrichmentMap networks were constructed for the darkgrey and lightgreen modules, two longest lists from Table 4. These are shown in Figure 2 and Additional file 2: Figure S3, respectively. The darkgrey module network shows multiple highly-connected nodes related to viral response. An EnrichmentMap network was also produced using the GSEA results for the set of genes nominally associated (p < 0.05) with the exacerbations phenotype (model #1); the GSEA results are provided in Additional file 2: Table S15 and the EnrichmentMap network is shown in Additional file 3: Figure S4. Many similar immune cell gene sets are seen, along with multiple non-specific results such as cancer gene sets.

**Table 4:** **GSEA results for modules associated with exacerbations phenotypes (gene sets with FDR q-value < 0.25 are shown and viral response gene sets are in bold)**

**Module name**	**Gene set description**	**ID**	**Set overlap**	**P-value**	**FDR q-value**
White (negative)*	NAIVE_BCELL_VS_MONOCYTE_UP	GSE22886	15	0.084	0.207
	CD4_TCELL_VS_BCELL_DN	GSE10325	18	0.122	0.230
	**BCELL_VS_MONOCYTE_DAY7_FLU_VACCINE_P**	**GSE29618**	17	0.185	0.216
Royalblue (positive)*	NAIVE_VS_PD1LOW_CD8_TCELL_DN	GSE26495	28	0.004	0.048
	**HEALTHY_VS_RSV_INF_INFANT_PBMC_UP**	**GSE34205**	15	0.016	0.062
	DEURIG_T_CELL_PROLYMPHOCYTIC_LEUKEMIA_DN		18	0.055	0.151
Lightgreen (negative)*	BCELL_VS_MDC_DAY7_FLU_VACCINE_UP	GSE29618	17	0.006	0.095
	CD4_TCELL_VS_BCELL_DN	GSE10325	19	0.012	0.05
	BCELL_VS_MDC_UP	GSE29618	15	0.012	0.043
	LUPUS_CD4_TCELL_VS_LUPUS_BCELL_DN	GSE10325	20	0.01	0.046
	DODD_NASOPHARYNGEAL_CARCINOMA_UP		15	0.023	0.068
	BCELL_VS_MONOCYTE_DAY7_FLU_VACCINE_UP	GSE29618	21	0.025	0.064
	BCELL_VS_MYELOID_UP	GSE10325	15	0.045	0.064
	NAIVE_BCELL_VS_MONOCYTE_UP	GSE22886	17	0.06	0.078
	BCELL_VS_MONOCYTE_UP	GSE29618	23	0.075	0.121
	CAGGTG_V$E12_Q6		16	0.116	0.149
	MEMORY_CD4_TCELL_VS_BCELL_DN	GSE3982	17	0.137	0.14
Darkturquoise (positive)*	NAIVE_VS_PD1LOW_CD8_TCELL_DN	GSE26495	26	0.057	0.090
	NAIVE_VS_PD1HIGH_CD8_TCELL_DN	GSE26495	19	0.158	0.166
Darkgrey (positive)*	CTRL_VS_NEWCASTLE_VIRUS_DC_8H_DN	GSE18791	30	0.000	0.012
	**BOSCO_INTERFERON_INDUCED_ANTIVIRAL_MODULE**		18	**0.003**	**0.038**
	UNSTIM_VS_4H_LPS_DC_TRANSLATED_RNA_DN	GSE14000	23	0.002	0.030
	MOSERLE_IFNA_RESPONSE		17	0.011	0.104
	TAKEDA_TARGETS_OF_NUP98_HOXA9_FUSION_10D_UP		20	0.016	0.094
	UNSTIM_VS_NEWCATSLE_VIRUS_DC_18H_DN	GSE18791	19	0.013	0.093
	UNSTIM_VS_NEWCATSLE_VIRUS_DC_6H_DN	GSE18791	31	0.031	0.105
	CTRL_VS_NEWCASTLE_VIRUS_DC_10H_DN	GSE18791	21	0.013	0.095
	CTRL_VS_NEWCASTLE_VIRUS_DC_12H_DN	GSE18791	19	0.026	0.134
	UNSTIM_VS_4H_LPS_DC_DN	GSE14000	24	0.031	0.141
	UNSTIM_VS_NEWCATSLE_VIRUS_DC_10H_DN	GSE18791	26	0.034	0.161
	CTRL_VS_NEWCASTLE_VIRUS_DC_4H_DN	GSE18791	22	0.052	0.166
	CTRL_VS_NEWCASTLE_VIRUS_DC_6H_DN	GSE18791	27	0.054	0.170
	BCELL_VS_LUPUS_BCELL_DN	GSE10325	17	0.094	0.250
	HECKER_IFNB1_TARGETS		21	0.102	0.246

*Positive or negative correlation between module and phenotype.

### Biomarker association with exacerbations phenotype

The association between the number of exacerbations and each of the plasma biomarkers was tested adjusting for covariates and the top ten results are provided in Table 5. Myeloperoxidase (MPO) was the top result (FDR q-value = 0.096), while club cell-16 (CC-16) showed nominal association (p < 0.05).

**Table 5:** **Biomarker association with exacerbation phenotype**

**Biomarker**	**Beta**	**Standard error**	**P-Value**	**FDR q-value**
MPO	0.33	0.096	0.00069	0.096
CC_16	0.20	0.098	0.041	0.98
ET1_Z	−0.44	0.25	0.074	0.98
HPT	0.16	0.094	0.086	0.98
ENA78	0.18	0.11	0.094	0.98
TNFB	−0.32	0.20	0.11	0.98
TP	−0.16	0.10	0.12	0.98
TSH	0.15	0.10	0.13	0.98
APOH	−0.15	0.10	0.14	0.98
TGFB3	0.30	0.20	0.14	0.98

## Discussion

In order to identify genes associated with COPD-related phenotypes, we initially used standard gene expression models. Despite the sample size, we found a lack of significant results, and a lack of specificity with respect to pathways or diseases from the enrichment analyses. However, network methods were able to group similar probes into correlated modules with potential common functions, that were then tested for association with phenotypes of interest. Using these methods we were able to observe immunological functions across the modules associated with acute exacerbations of COPD. In addition, we were able to recapitulate the molecular signatures of previous studies involving viral lung infections and exacerbations of asthma, a related respiratory disease. The ability to uncover such signatures is limited given that only subjects having two or fewer exacerbations within the previous year were included in the study, as a wider range of exacerbation frequencies would provide greater resolution to detect the molecular underpinnings of COPD exacerbations. Yet the network methods added value to address these limitations of the dataset.

WGCNA allowed for identification of the putative hub genes for each of the five modules associated with exacerbations. These hubs include the immunoglobulin heavy constant mu (IGHM) gene in the white module, that codes for the C region of the mu heavy chain (defines the IgM isotype). Lending to its role in lung disease, IGHM was found to be down-regulated in idiopathic pulmonary fibrosis (IPF) whole blood samples versus normal samples [9]. From the royalblue module, the putative hub was found to be KLRD1 (CD94) that codes for an antigen preferentially expressed on NK cells. A decrease in its expression has been observed in the blood of COPD patients versus controls [25]. The gene product for AFF3 from the lightgreen module is a tissue restricted nuclear transcriptional activator, formerly known as lymphoid nuclear protein related to AF4, and it was down-regulated with respect to controls in BEAS-2B cells (immortalized human bronchial epithelial cells) treated with low doses of cigarette smoke condensate [26]. From the darkturquoise module, GPR56 encodes G protein-coupled receptor 56; its involvement in COPD has not been previously established. Lastly, the hubs from the darkgrey module, GBP1 and GBP5, guanylate binding proteins whose expression is induced by interferon and are markers of M1 macrophage population, were found to be down-regulated by cigarette smoke in monocyte-derived macrophages [27]. Taken as a whole, these hubs lend additional evidence to the specific role of each module: B cell activity for the white and lightgreen modules, NK cell activity for the royalblue module, and macrophage involvement in the darkgrey module.

GO and KEGG pathway enrichment analysis provided common functions of the module genes, namely the immune system response to bacterial and viral infections. Additionally, the significant enrichment for B cell and asthma pathways in the white module are of note, as are the cytokine and viral response related enrichments in the darkturquoise and darkgrey modules. However, the GSEA results were more informative, revealing signatures of gene expression previously identified in studies involving lung infections, which is consistent with the infectious etiology of most COPD exacerbations. One example was a top result for the darkgrey module (Table 4). The gene set, BOSCO_INTERFERON_INDUCED_ANTIVIRAL_MODULE, was a WGCNA module (n = 78 genes) composed of genes downstream of interferon signaling, that was associated with lung function in a study of sputum samples of children with acute asthma exacerbations [28]. This study found decreased activation of interferon pathways in asthmatic children with chronic airway obstruction compared with those without airway obstruction. The darkgrey module may harbor genes associated with differential responses during exacerbations that perhaps also play a role in onset, frequency and severity of COPD exacerbations.

In another example, the royalblue module enrichment results include the gene set GSE34205_HEALTHY_VS_RSV_INF_INFANT_PBMC_UP, genes differentially expressed in blood samples from healthy infants and infants suffering from respiratory syncytial virus (RSV) infection [29]. RSV is a major respiratory pathogen that is more common in infants; however the elderly and patients with COPD or other lung diseases are also at risk of developing RSV infections [2]. Using network analysis methods and GSEA, we were able to identify modules that contain genes demonstrating a signature previously seen in the sputum of patients suffering from acute asthma exacerbations and the blood of patients with respiratory infections. However, we detected these signatures using expression data from whole blood of COPD patients, collected months following exacerbations.

The EnrichmentMap network of GSEA results for the darkgrey module (Figure 2) shows an overlap between the asthma exacerbation [28] and the viral-response gene sets, as would be expected given the virus-induced nature of many exacerbations. However the relatively narrow widths of many edges demonstrate that these gene sets are largely distinct. In the network for the lightgreen module in Additional file 2: Figure S3, relatively strong connections exist between the nodes, demonstrating an enrichment of B cell pathway activity within the gene sets. In contrast to the specificity of the networks for the darkgrey and lightgreen modules, the EnrichmentMap for the probe-based analysis gene set shown in Additional file 2: Figure S4 is less tightly connected with many nodes having zero connections. The probe-based analysis is less specific than the module analyses. Although the red sub-network in Additional file 2: Figure S4 contains several viral response results, the blue sub-network contains various cancer gene sets, in addition to infectious diseases.

In the biomarker analysis, we found an association between MPO and the number of exacerbations, persisting months following illness. Serum MPO levels have been shown to be increased in COPD patients during acute exacerbations [30]. However in contrast to our finding, a previous study did not find a difference in sputum MPO levels between patients with stable COPD having frequent (>3/year) or infrequent (<2/year) exacerbations in the previous 12 months [31]. In addition, a study of bronchoalveolar lavage samples did not find significantly higher MPO levels in COPD patients with frequent (≥3/year) exacerbations as compared to infrequent (<3/year) exacerbations [32]. Perhaps the peripheral blood based signature of COPD exacerbations is able to persist due to systemic modulation of the immune system. Longitudinal studies have shown a reduction in serum MPO levels during treatment for acute exacerbations of chronic bronchitis, followed by the return to higher levels following treatment [33], however no association with the number of exacerbations or their severity was noted. Taken together, our finding and the previous studies provide evidence for MPO as part of the molecular signature to be considered when predicting the onset, severity and frequency of future COPD exacerbations, as it has been previously established as an inflammatory marker, and specifically in COPD [34].

The use of gene expression data from whole blood instead of lung tissue samples may limit the ability of our approach to detect signatures in the specific disease tissue. However, peripheral blood is more easily accessed than lung tissue and may provide specific insight into the infectious, immune and inflammatory mechanisms of COPD exacerbations. Like lung tissue, whole blood is a complex mixture of different cell types. Complete blood counts (CBC) with cell differentials are routinely measured in clinical practice, but were not available in the TESRA study. However, our analysis was able to distinguish B and T cell signatures, cell types which would not be discriminated in a standard CBC. An additional limitation of our approach involves the use biomarker and gene expression data as markers of exacerbations in the past year, yet this may indicate a stable molecular signature differing from that of lung function and other markers of severity. Lastly, one potential confounder is the external trigger of the exacerbation. Regardless of an individual’s genetic predisposition for exacerbations, a viral, bacterial or other environmental cause may lead to different signatures. Despite these limitations, we were able to effectively uncover expression signatures of frequent COPD exacerbations in peripheral whole blood samples.

## Conclusions

In this study a distinct signature of COPD exacerbations was observed in peripheral whole blood months following the illness. This signature was correlated with previous studies involving exacerbations in asthma and viral lung infections, and therefore, it may be useful in uncovering the molecular pathogenesis of COPD exacerbations. The moderate association of MPO and number of exacerbations adds to the body of information supporting the use of MPO as a biomarker. Future studies with longitudinal expression data would allow an investigation of the temporal nature of the network modules, separating causal modules from the downstream modules demonstrating expression changes as a result of the disease state. Similarly, the methods presented here would benefit from gene expression data obtained during acute exacerbations, in addition to samples obtained months following the illness, particularly with respect to prediction. Overall, these approaches will help to elucidate the genetic component determining the heterogeneity of onset, severity, and frequency of exacerbations seen in COPD patients.

## Additional files

Additional file 1:
**Institutional Review Boards for TESRA trial sites.**
Additional file 2: Table S1. Expression data cleaning process. **Table S2.** Regression models for association with outcomes of interest. **Table S3.** Top five results from test of gene expression association with phenotype variables. **Figure S1.** Clustering dendrograms based on topological overlap in WGCNA (block #1). **Figure S2.** Clustering dendrograms based on topological overlap in WGCNA (block #2). **Table S4.** WGCNA modules. **Table S5.** Module eigengene association with exacerbations phenotypes. **Table S6.** White module contents and information. **Table S7.** Royalblue module contents and information. **Table S8.** Lightgreen module contents and information. **Table S9.** Darkturquoise module contents and information. **Table S10.** Darkgrey module contents and information. **Table S11.** Top pathway analysis results for royalblue module. **Table S12.** Top pathway analysis results for lightgreen module. **Table S13.** Top pathway analysis results for darkturquoise module. **Table S14.** Top pathway analysis results for darkgrey module.**Figure S3.** EnrichmentMap network for GSEA results from lightgreen module. Nodes correspond to gene sets and edges correspond to an overlap of the genes within two sets. **Table S15.** GSEA results for probe association with exacerbations phenotype. Additional file 3: Figure S4. EnrichmentMap for GSEA results for probe association with exacerbation phenotype.
