# Supplementary material for: Identifying a gene expression signature of frequent COPD exacerbations in peripheral blood using network methods
Source: BMC Med Genomics. 2015 Jan 13;8:1. doi: 10.1186/s12920-014-0072-y (PMC4302028; doi:10.1186/s12920-014-0072-y)
Supplement: Additional file 2: Table S1. — Expression data cleaning process. Table S2. Regression models for association with outcomes of interest. Table S3. Top five results from test of gene expression association with phenotype variables. Figure S1. Clustering dendrograms based on topological overlap in WGCNA (block #1). Figure S2. Clustering dendrograms based on topological overlap in WGCNA (block #2). Table S4. WGCNA modules. Table S5. Module eigengene association with exacerbations phenotypes. Table S6. White module contents and information. Table S7. Royalblue module contents and information. Table S8. Lightgreen module contents and information. Table S9. Darkturquoise module contents and information. Table S10. Darkgrey module contents and information. Table S11. Top pathway analysis results for royalblue module. Table S12. Top pathway analysis results for lightgreen module. Table S13. Top pathway analysis results for darkturquoise module. Table S14. Top pathway analysis results for darkgrey module.Figure S3. EnrichmentMap network for GSEA results from lightgreen module. Nodes correspond to gene sets and edges correspond to an overlap of the genes within two sets. Table S15. GSEA results for probe association with exacerbations phenotype. [file 12920_2014_72_MOESM2_ESM.doc]

**Supplemental material**

Table S1. Expression data cleaning process

| Study population | 410 |
| --- | --- |
| Total number of gene expression arrays available | 309 |
| Sample replicates within the data | -39 |
| Arrays passed to quality control | 270 |
| Failed quality control based on multiple quality metrics | -3 |
| Passed quality control | 267 |
| Subjects self-identified as other than Caucasian | -18 |
| Samples available for analysis | 249 |
| Subjects excluded based on ancestry not clustered with Caucasians (determined using genotype data) | 1 |
|  |  |
| **Final number of samples/arrays for analysis** | **248** |

Expression association with phenotype variables:

Table S21. Regression models for association with outcomes of interest

| **Model #** | **Variable of interest** | **Model** |
| --- | --- | --- |
| 1 | Exacerbations in past 12 months | EXP ~ **EXACERB** + AGE + SEX + pack-years + FEV1.PP + Lot_number |
| 2 | Exacerbations in past 12 months  in binary format:  1 = none, 2 = 1 or 2 | EXP ~ **EXACERB01p** + AGE + SEX + pack-years + FEV1.PP + Lot_number |
| 3 | FEV1 (L) | EXP ~ **FEV1** + AGE + SEX + Height + pack-years + Lot_number |
| 4 | FEV1/FVC | EXP ~ **FEV1FVC** + AGE + SEX + Height + pack-years + Lot_number |
| 5 | % LAA ≤-910 HU | EXP ~ **LAA910** + AGE + SEX + BMI + pack-years + Lot_number |
| 6 | Perc 15 | EXP ~ **Perc15** + AGE + SEX + BMI + pack-years + Lot_number |
| 7 | TLC (L) | EXP ~ **TLC** + AGE + SEX + Height + pack-years + Lot_number |
| 8 | TLC % | EXP ~ **TLC.PP** + pack-years + Lot_number |
| 9 | DLCO (mL/min/mmHg) | EXP ~ **DLCO** + AGE + SEX + Height + pack-years + Lot_number |
| 10 | Residual Volume % | EXP ~ **RV.PP** + pack-years + Lot_number |
| 11 | Inspiratory Capacity | EXP ~ **IC** + AGE + SEX + Height + pack-years + Lot_number |
| 12 | 6 Minute Walk Test (m) | EXP ~ **6MWT** + AGE + SEX + Height + pack-years + Lot_number |
| definitions: .PP = % predicted, EXP = expression values | | |

Table S3. Top five results from test of gene expression association with phenotype variables (see Table S2 for variable definitions and models).

| **model** | **probe** | **Log(fold change)** | **p-value** | **FDR q-value** | **Gene symbol** |
| --- | --- | --- | --- | --- | --- |
| **EXACERB**  model #1  1338 total  results with  p-value < 0.05 | 1562790_at | 0.18197 | 2.02E-005 | 0.9395 | NCALD |
|  | 239017_at | -0.16939 | 3.44E-005 | 0.9395 | NA |
|  | 205643_s_at | 0.11466 | 5.93E-005 | 0.9999 | PPP2R2B |
|  | 244793_at | -0.14636 | 9.22E-005 | 0.9999 | NA |
|  | 243780_at | -0.2908 | 2.06E-004 | 0.9999 | NA |
| **EXACERB01p**  model #2  2222 total  results with  p-value < 0.05 | 239017_at | -0.22829 | 2.24E-005 | 0.9995 | NA |
|  | 1564003_at | 0.07654 | 1.49E-004 | 0.9995 | NA |
|  | 1562790_at | 0.21082 | 1.90E-004 | 0.9995 | NCALD |
|  | 239519_at | 0.15518 | 2.01E-004 | 0.9995 | NA |
|  | 243780_at | -0.37137 | 3.26E-004 | 0.9995 | NA |
| **FEV1**  model #3  3656 total  results with  p-value < 0.05 | 215897_at | -0.381 | 7.6E-006 | 0.4145 | MED25 |
|  | 223247_at | 0.2343 | 3.8E-005 | 0.582 | MED10 |
|  | 218712_at | 0.411 | 4.6E-005 | 0.582 | C1orf109 |
|  | 238106_at | -0.3275 | 6.3E-005 | 0.582 | NA |
|  | 240254_at | 0.4874 | 7.40E-005 | 0.582 | NA |
| **FEV1/FVC**  model #4  3105 total  results with  p-value < 0.05 | 219117_s_at | 1.037 | 2.73E-005 | 0.5171 | FKBP11 |
|  | 206366_x_at | 1.4895 | 3.57E-005 | 0.5171 | XCL1 |
|  | 214567_s_at | 1.685 | 3.58E-005 | 0.5171 | NA |
|  | 212009_s_at | 1.2234 | 4.30E-005 | 0.5171 | STIP1 |
|  | 213840_s_at | 0.8695 | 4.73E-005 | 0.5171 | MRPS12 |
| **LAA910**  model #5  1737 total  results with  p-value < 0.05 | 236540_at | -0.007554 | 5.03E-005 | 0.9861 | NA |
|  | 225255_at | -0.007047 | 7.35E-005 | 0.9861 | MRPL35 |
|  | 205425_at | 0.0104 | 3.41E-004 | 0.9861 | HIP1 |
|  | 212394_at | 0.005077 | 3.42E-004 | 0.9861 | EMC1 |
|  | 232418_at | 0.003645 | 3.64E-004 | 0.9861 | LZTFL1 |
| **Perc15**  model #6  1564 total  results with  p-value < 0.05 | 219117_s_at | 0.004313 | 1.17E-005 | 0.3774 | FKBP11 |
|  | 236540_at | 0.005466 | 1.38E-005 | 0.3774 | NA |
|  | 1559722_at | -0.004461 | 3.81E-005 | 0.4567 | NA |
|  | 225255_at | 0.004924 | 4.16E-005 | 0.4567 | MRPL35 |
|  | 210377_at | -0.00303 | 4.18E-005 | 0.4567 | ACSM3 |
| **TLC (L)**  model #7  1190 total  results with  p-value < 0.05 | 231012_at | 0.08459 | 0.0001449 | 1 | SLC35G1 |
|  | 1556581_at | -0.07891 | 0.0004272 | 1 | ZNF778 |
|  | 216728_at | 0.03053 | 0.0007745 | 1 | NA |
|  | 1561916_at | 0.05505 | 0.0007785 | 1 | NA |
|  | 244488_at | 0.07815 | 0.0010315 | 1 | LSM14B |
| **TLC.PP**  model #8  3415 total  results with  p-value < 0.05 | 227229_at | -0.005699 | 1.35E-005 | 0.4109 | VPS53 |
|  | 205224_at | -0.004682 | 1.50E-005 | 0.4109 | SURF2 |
|  | 226489_at | 0.006932 | 1.17E-004 | 0.734 | TMCC3 |
|  | 91816_f_at | -0.007919 | 1.24E-004 | 0.734 | MEX3D |
|  | 218734_at | -0.00642 | 2.06E-004 | 0.734 | NAA40 |
| **DLCO**  model #9  2372 total  results with  p-value < 0.05 | 233182_x_at | 0.02972 | 2.28E-005 | 0.6917 | ATXN3 |
|  | 1553801_a_at | 0.03169 | 2.53E-005 | 0.6917 | DTD2 |
|  | 1557196_a_at | 0.02879 | 6.38E-005 | 0.9991 | NA |
|  | 235883_at | 0.03064 | 1.01E-004 | 0.9991 | NA |
|  | 204994_at | -0.03722 | 1.23E-004 | 0.9991 | MX2 |
| **RV.PP**  model #10  3484 total  results with  p-value < 0.05 | 1553518_at | -0.002601 | 9.15E-005 | 0.7116 | NA |
|  | 218638_s_at | -0.004866 | 9.34E-005 | 0.7116 | NA |
|  | 221222_s_at | 0.002824 | 1.54E-004 | 0.7116 | C1orf56 |
|  | 1559434_at | -0.001879 | 1.68E-004 | 0.7116 | NA |
|  | 235170_at | 0.003605 | 1.76E-004 | 0.7116 | ZNF92 |
| **IC**  model #11  3408 total  results with  p-value < 0.05 | 1557246_at | 0.2578 | 4.47E-005 | 0.8011 | KIDINS220 |
|  | 207291_at | -0.241 | 1.17E-004 | 0.8011 | PRRG4 |
|  | 1555397_at | -0.2106 | 1.35E-004 | 0.8011 | MYO1D |
|  | 1563541_at | -0.1062 | 1.76E-004 | 0.8011 | NA |
|  | 1557727_at | -0.2946 | 1.82E-004 | 0.8011 | PCBP1-AS1 |
| **6MWT**  model #12  2867 total  results with  p-value < 0.05 | 233249_at | 0.0010508 | 4.75E-007 | 0.02597 | NA |
|  | 232034_at | -0.0012018 | 2.17E-005 | 0.39545 | LINC00537 |
|  | 1555898_at | 0.0008671 | 2.69E-005 | 0.39545 | ANKRD36C |
|  | 206336_at | 0.0006641 | 3.40E-005 | 0.39545 | CXCL6 |
|  | 213974_at | 0.000517 | 3.62E-005 | 0.39545 | ADAMTSL3 |

Network analysis:

Figure S1. Clustering dendrograms based on topological overlap in WGCNA (block #1)


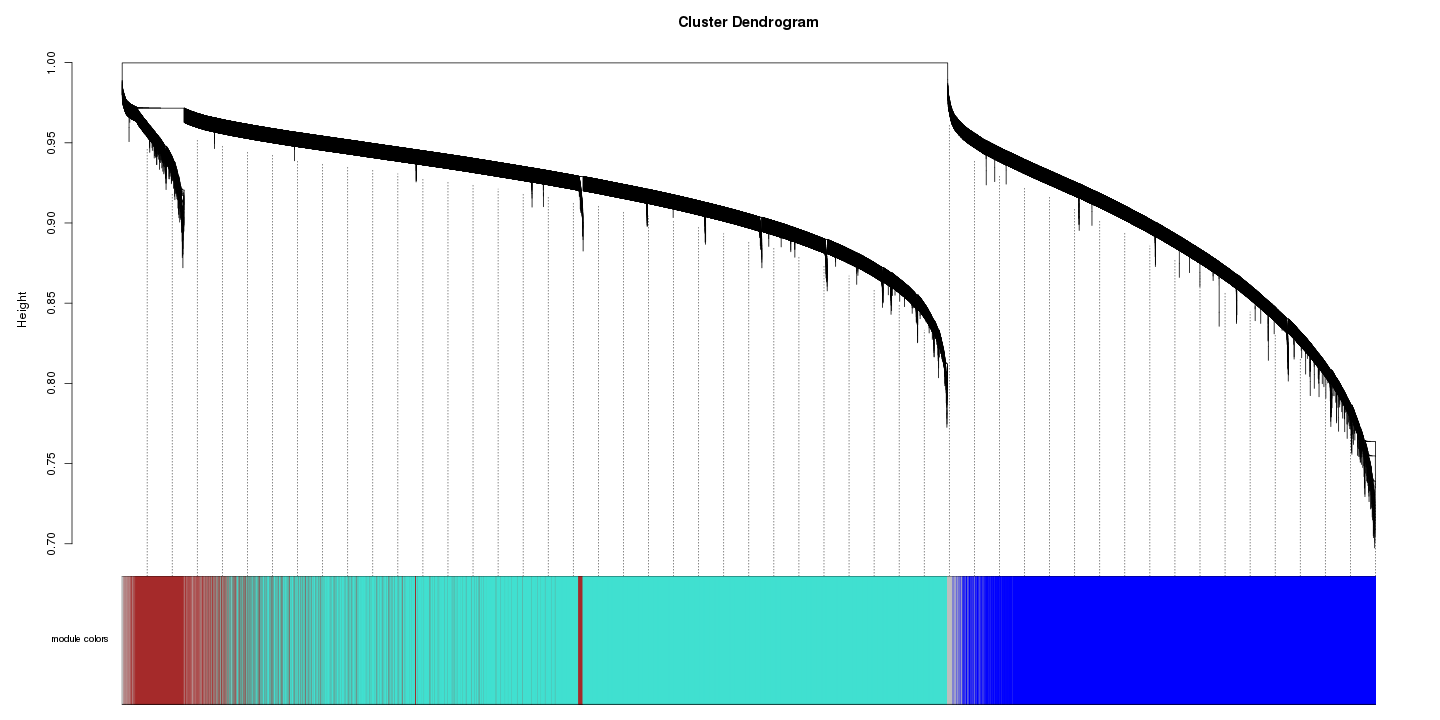


Figure S2. Clustering dendrograms based on topological overlap in WGCNA (block #2)


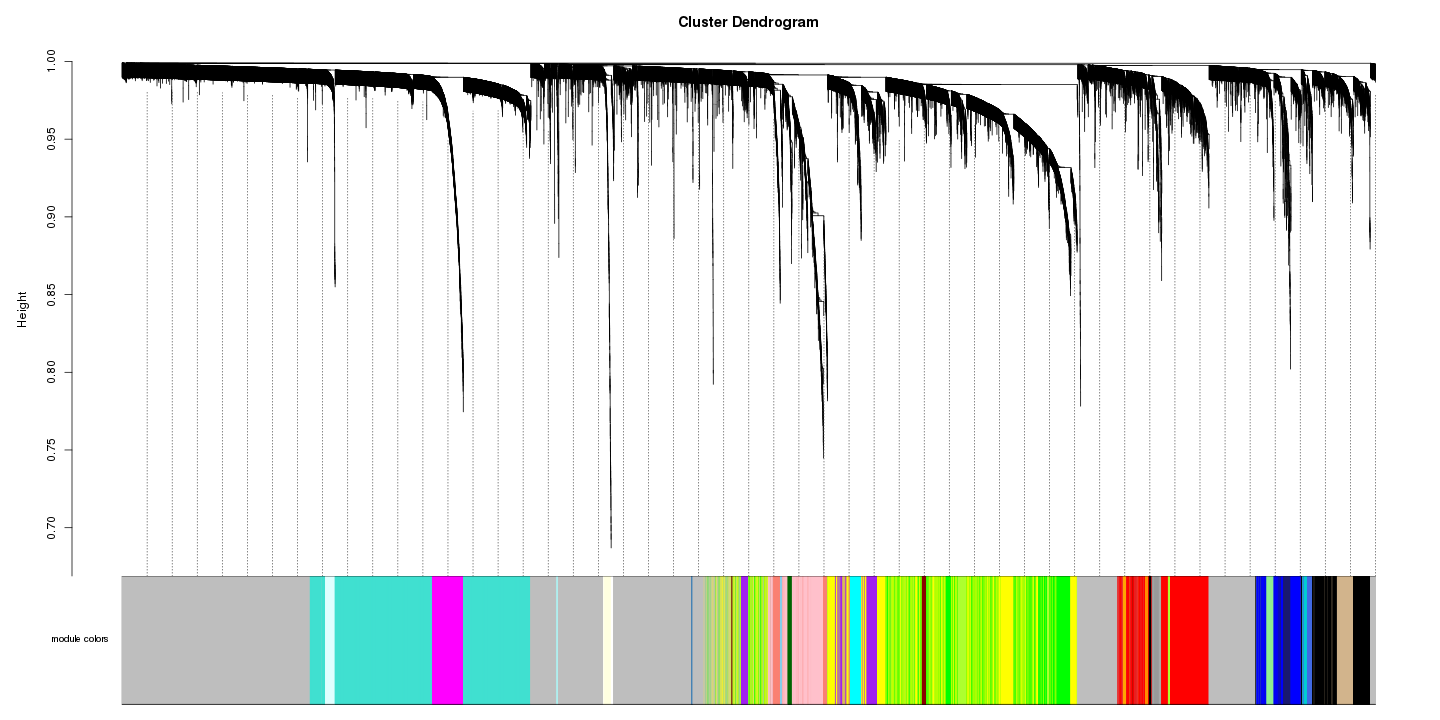


Table S4. WGCNA modules (highlighted - association with exacerbations phenotypes)

| **Module name** | **Minimum module self cor** | **Mean module self cor** | **SEM module self cor** | **Module size** |
| --- | --- | --- | --- | --- |
| lightcyan | 0.41600 | 0.62900 | 0.00542 | 184 |
| magenta | 0.25800 | 0.63200 | 0.00656 | 612 |
| turquoise | 0.00504 | 0.63300 | 0.00110 | 19748 |
| lightyellow | 0.30200 | 0.70700 | 0.01590 | 140 |
| white | 0.44900 | 0.71800 | 0.01520 | 57 |
| cyan | 0.45500 | 0.70300 | 0.00703 | 216 |
| purple | 0.38400 | 0.63100 | 0.00441 | 498 |
| brown | 0.19100 | 0.62900 | 0.00237 | 3019 |
| green | 0.06930 | 0.58500 | 0.00395 | 1747 |
| yellow | 0.11800 | 0.53600 | 0.00292 | 2824 |
| paleturquoise | 0.41800 | 0.75300 | 0.02220 | 30 |
| midnightblue | 0.25700 | 0.66200 | 0.01020 | 207 |
| saddlebrown | 0.45700 | 0.66800 | 0.01990 | 33 |
| darkturquoise | 0.46400 | 0.67300 | 0.01150 | 73 |
| royalblue | 0.33300 | 0.68100 | 0.00992 | 118 |
| blue | 0.17400 | 0.66300 | 0.00159 | 10294 |
| darkorange | 0.54500 | 0.69300 | 0.01120 | 62 |
| lightgreen | 0.45900 | 0.65100 | 0.00960 | 147 |
| black | 0.12500 | 0.55000 | 0.00362 | 903 |
| tan | 0.41700 | 0.63100 | 0.00473 | 344 |
| salmon | 0.36100 | 0.73000 | 0.00731 | 269 |
| skyblue | 0.61100 | 0.77400 | 0.01060 | 48 |
| darkgreen | 0.61900 | 0.79300 | 0.00793 | 86 |
| pink | 0.27100 | 0.66400 | 0.00608 | 763 |
| orange | 0.45600 | 0.64200 | 0.01070 | 64 |
| steelblue | 0.55600 | 0.68100 | 0.01420 | 32 |
| darkgrey | 0.64900 | 0.80600 | 0.00845 | 65 |
| grey60 | 0.29500 | 0.65100 | 0.01340 | 171 |
| red | 0.25700 | 0.57300 | 0.00349 | 1313 |
| darkred | 0.54100 | 0.71400 | 0.00742 | 87 |
| greenyellow | 0.26400 | 0.63300 | 0.00665 | 395 |
| grey | 0.00001 | 0.17900 | 0.00124 | 10126 |

Table S5. Module eigengene association with exacerbations phenotypes (highlighted - association with exacerbations phenotypes)

| **Module name** | **Exacerbations – continuous (0,1,2)** | | **Exacerbations – binary (0 vs. 1+)** | |
| --- | --- | --- | --- | --- |
|  | **P.Value** | **FDR q-value** | **P.Value** | **FDR q-value** |
| white | 0.00071 | 0.023 | 0.00077 | 0.025 |
| royalblue | 0.032 | 0.32 | 0.086 | 0.69 |
| lightgreen | 0.033 | 0.32 | 0.046 | 0.49 |
| darkturquoise | 0.04 | 0.32 | 0.19 | 0.70 |
| darkgrey | 0.0715 | 0.46 | 0.022 | 0.35 |
| paleturquoise | 0.21 | 0.97 | 0.24 | 0.70 |
| salmon | 0.26 | 0.97 | 0.16 | 0.70 |
| grey60 | 0.32 | 0.97 | 0.19 | 0.70 |
| pink | 0.43 | 0.97 | 0.19 | 0.70 |
| lightyellow | 0.47 | 0.97 | 0.33 | 0.70 |
| skyblue | 0.49 | 0.97 | 0.28 | 0.70 |
| steelblue | 0.52 | 0.97 | 0.35 | 0.70 |
| magenta | 0.53 | 0.97 | 0.84 | 0.90 |
| cyan | 0.56 | 0.97 | 0.20 | 0.70 |
| saddlebrown | 0.65 | 0.97 | 0.50 | 0.72 |
| tan | 0.67 | 0.97 | 0.44 | 0.72 |
| turquoise | 0.71 | 0.97 | 0.43 | 0.72 |
| purple | 0.71 | 0.97 | 0.31 | 0.70 |
| grey | 0.74 | 0.97 | 0.46 | 0.72 |
| midnightblue | 0.76 | 0.97 | 0.75 | 0.90 |
| orange | 0.78 | 0.97 | 0.64 | 0.86 |
| darkgreen | 0.79 | 0.97 | 0.29 | 0.70 |
| brown | 0.82 | 0.97 | 0.65 | 0.86 |
| red | 0.82 | 0.97 | 0.97 | 0.97 |
| yellow | 0.83 | 0.97 | 0.35 | 0.70 |
| greenyellow | 0.84 | 0.97 | 0.83 | 0.90 |
| darkorange | 0.85 | 0.97 | 0.81 | 0.90 |
| blue | 0.85 | 0.97 | 0.83 | 0.90 |
| darkred | 0.89 | 0.97 | 0.37 | 0.70 |
| black | 0.94 | 0.97 | 0.48 | 0.72 |
| green | 0.94 | 0.97 | 0.67 | 0.86 |
| lightcyan | 0.998 | 0.998 | 0.90 | 0.93 |

Table S6. White module contents and information. Hub gene is highlighted.

| **Module information** | **Probe** | **GS (p-value)** | **MM (module cor.)** | **Gene symbol** |
| --- | --- | --- | --- | --- |
| Module name: white  Number of probes: 57  Minimum self correlation: 0.45  Mean self correlation: 0.72 | 243780_at | 0.0002057 | 0.833 | NA |
|  | 39318_at | 0.0008487 | 0.852 | TCL1A |
|  | 212827_at | 0.001628 | 0.896 | IGHM |
|  | 205901_at | 0.001675 | 0.695 | PNOC |
|  | 219737_s_at | 0.001775 | 0.605 | PCDH9 |
|  | 204581_at | 0.001809 | 0.858 | CD22 |
|  | 1559618_at | 0.001921 | 0.471 | LOC100129447 |
|  | 205297_s_at | 0.002189 | 0.741 | CD79B |
|  | 224404_s_at | 0.00226 | 0.644 | FCRL5 |
|  | 230983_at | 0.002619 | 0.896 | FAM129C |
|  | 209995_s_at | 0.00283 | 0.854 | TCL1A |
|  | 215621_s_at | 0.003274 | 0.731 | NA |
|  | 1555799_at | 0.003428 | 0.602 | FCRL5 |
|  | 242104_at | 0.003599 | 0.846 | NA |
|  | 1561086_at | 0.003675 | 0.793 | NA |
|  | 226878_at | 0.003786 | 0.761 | HLA-DOA |
|  | 221969_at | 0.003871 | 0.892 | PAX5 |
|  | 235400_at | 0.003957 | 0.840 | FCRLA |
|  | 221239_s_at | 0.005144 | 0.812 | FCRL2 |
|  | 241577_at | 0.005305 | 0.765 | NA |
|  | 243613_at | 0.005656 | 0.500 | DENND5B |
|  | 232286_at | 0.005784 | 0.775 | NA |
|  | 205049_s_at | 0.00616 | 0.873 | CD79A |
|  | 222285_at | 0.007187 | 0.796 | NA |
|  | 239214_at | 0.007212 | 0.554 | LOC100130458 |
|  | 1565034_s_at | 0.007354 | 0.804 | AFF3 |
|  | 209582_s_at | 0.007462 | 0.766 | CD200 |
|  | 206126_at | 0.008507 | 0.767 | CXCR5 |
|  | 205735_s_at | 0.008911 | 0.784 | AFF3 |
|  | 226122_at | 0.009471 | 0.598 | PLEKHG1 |
|  | 1555779_a_at | 0.01069 | 0.856 | CD79A |
|  | 224406_s_at | 0.01073 | 0.602 | FCRL5 |
|  | 206760_s_at | 0.01361 | 0.761 | FCER2 |
|  | 224193_s_at | 0.01814 | 0.788 | FCRL2 |
|  | 38521_at | 0.02509 | 0.851 | CD22 |
|  | 215925_s_at | 0.02948 | 0.722 | CD72 |
|  | 236307_at | 0.03148 | 0.622 | NA |
|  | 236163_at | 0.03287 | 0.449 | LIX1 |
|  | 1553369_at | 0.03793 | 0.793 | FAM129C |
|  | 1566879_at | 0.04601 | 0.696 | NA |
|  | 206530_at | 0.04652 | 0.551 | RAB30 |
|  | 207819_s_at | 0.04989 | 0.628 | ABCB4 |
|  | 224026_at | 0.05329 | 0.713 | NA |
|  | 203796_s_at | 0.05785 | 0.709 | BCL7A |
|  | 211011_at | 0.06482 | 0.645 | COL19A1 |
|  | 210448_s_at | 0.06555 | 0.626 | P2RX5 |
|  | 244696_at | 0.07842 | 0.763 | NA |
|  | 243967_at | 0.08232 | 0.676 | AFF3 |
|  | 232204_at | 0.08486 | 0.534 | EBF1 |
|  | 219301_s_at | 0.08524 | 0.615 | CNTNAP2 |
|  | 230877_at | 0.09704 | 0.794 | NA |
|  | 205671_s_at | 0.1254 | 0.586 | HLA-DOB |
|  | 223568_s_at | 0.1322 | 0.678 | PPAPDC1B |
|  | 223522_at | 0.1482 | 0.638 | NA |
|  | 223569_at | 0.1719 | 0.625 | PPAPDC1B |
|  | 217422_s_at | 0.2171 | 0.751 | CD22 |
|  | 244172_at | 0.2956 | 0.646 | NA |

Table S7. Royalblue module contents and information. Hub genes are highlighted.

| **Module information** | **Probe** | **GS (p-value)** | **MM (module cor.)** | **Gene symbol** |
| --- | --- | --- | --- | --- |
| Module name: royalblue  Number of probes: 118  Minimum self correlation: 0.33  Mean self correlation: 0.68 | 1560573_at | 0.0004367 | 0.571 | LINC00944 |
|  | 243905_at | 0.0006656 | 0.583 | LINC00944 |
|  | 210865_at | 0.0021 | 0.584 | FASLG |
|  | 202458_at | 0.003325 | 0.755 | PRSS23 |
|  | 210606_x_at | 0.005767 | 0.891 | KLRD1 |
|  | 205257_s_at | 0.006149 | 0.531 | AMPH |
|  | 207795_s_at | 0.007002 | 0.877 | KLRD1 |
|  | 212509_s_at | 0.009688 | 0.637 | MXRA7 |
|  | 244764_at | 0.01197 | 0.588 | HIVEP3 |
|  | 206170_at | 0.01404 | 0.708 | ADRB2 |
|  | 203215_s_at | 0.01881 | 0.710 | MYO6 |
|  | 220613_s_at | 0.02064 | 0.812 | SYTL2 |
|  | 234658_at | 0.02611 | 0.713 | NA |
|  | 222860_s_at | 0.02738 | 0.751 | PDGFD |
|  | 205758_at | 0.03243 | 0.755 | CD8A |
|  | 223836_at | 0.03352 | 0.763 | FGFBP2 |
|  | 210321_at | 0.03394 | 0.707 | GZMH |
|  | 211494_s_at | 0.03432 | 0.662 | SLC4A4 |
|  | 232914_s_at | 0.03769 | 0.751 | SYTL2 |
|  | 233379_at | 0.04221 | 0.801 | PRR5L |
|  | 219304_s_at | 0.04356 | 0.606 | PDGFD |
|  | 216191_s_at | 0.04467 | 0.677 | NA |
|  | 207796_x_at | 0.04602 | 0.670 | KLRD1 |
|  | 204205_at | 0.04839 | 0.704 | APOBEC3G |
|  | 215937_at | 0.05007 | 0.781 | PTGDR |
|  | 213830_at | 0.05135 | 0.619 | YME1L1 |
|  | 238718_at | 0.05613 | 0.611 | NA |
|  | 217143_s_at | 0.05665 | 0.627 | YME1L1 |
|  | 231440_at | 0.05821 | 0.627 | LINC00943 |
|  | 226279_at | 0.0595 | 0.690 | PRSS23 |
|  | 231776_at | 0.06332 | 0.806 | EOMES |
|  | 222838_at | 0.06534 | 0.761 | SLAMF7 |
|  | 225525_at | 0.07252 | 0.680 | KIAA1671 |
|  | 205821_at | 0.07529 | 0.791 | NA |
|  | 223125_s_at | 0.07552 | 0.743 | C1orf21 |
|  | 204066_s_at | 0.07933 | 0.640 | AGAP1 |
|  | 1568795_at | 0.08109 | 0.641 | NA |
|  | 214061_at | 0.08169 | 0.605 | TBC1D31 |
|  | 226348_at | 0.08481 | 0.745 | NA |
|  | 211144_x_at | 0.08659 | 0.803 | NA |
|  | 212599_at | 0.08742 | 0.628 | AUTS2 |
|  | 220646_s_at | 0.08908 | 0.745 | KLRF1 |
|  | 206589_at | 0.08931 | 0.568 | GFI1 |
|  | 210690_at | 0.09204 | 0.827 | KLRC4 |
|  | 219383_at | 0.09367 | 0.794 | PRR5L |
|  | 225496_s_at | 0.09899 | 0.823 | SYTL2 |
|  | 1557985_s_at | 0.1015 | 0.786 | CEP78 |
|  | 215894_at | 0.1028 | 0.795 | PTGDR |
|  | 211597_s_at | 0.1058 | 0.787 | HOPX |
|  | 231875_at | 0.1071 | 0.741 | KIF21A |
|  | 236782_at | 0.1077 | 0.873 | SAMD3 |
|  | 239744_at | 0.1086 | 0.766 | NA |
|  | 230753_at | 0.1094 | 0.595 | PATL2 |
|  | 210288_at | 0.1097 | 0.751 | KLRG1 |
|  | 238419_at | 0.1209 | 0.725 | PHLDB2 |
|  | 236394_at | 0.1218 | 0.553 | A2MP1 |
|  | 1555040_at | 0.1234 | 0.696 | LINC00612 |
|  | 234165_at | 0.1262 | 0.733 | PTGDR |
|  | 217757_at | 0.1281 | 0.681 | A2M |
|  | 1555691_a_at | 0.1298 | 0.857 | NA |
|  | 209813_x_at | 0.1301 | 0.810 | NA |
|  | 213849_s_at | 0.1341 | 0.706 | PPP2R2B |
|  | 202679_at | 0.1403 | 0.749 | NPC1 |
|  | 204731_at | 0.1415 | 0.853 | TGFBR3 |
|  | 239104_at | 0.1444 | 0.632 | NA |
|  | 216298_at | 0.1619 | 0.525 | TARP |
|  | 1566607_at | 0.1626 | 0.797 | NA |
|  | 240413_at | 0.1729 | 0.818 | PYHIN1 |
|  | 239237_at | 0.1766 | 0.767 | TRG-AS1 |
|  | 231837_at | 0.1772 | 0.572 | USP28 |
|  | 238551_at | 0.1787 | 0.695 | FUT11 |
|  | 1553876_at | 0.181 | 0.651 | SAMD3 |
|  | 228774_at | 0.1898 | 0.720 | CEP78 |
|  | 207330_at | 0.1901 | 0.693 | PZP |
|  | 241206_at | 0.1975 | 0.706 | NA |
|  | 230110_at | 0.2044 | 0.610 | MCOLN2 |
|  | 205933_at | 0.2178 | 0.453 | SETBP1 |
|  | 216920_s_at | 0.2266 | 0.820 | NA |
|  | 226625_at | 0.2285 | 0.791 | TGFBR3 |
|  | 206785_s_at | 0.2372 | 0.619 | NA |
|  | 1552678_a_at | 0.2374 | 0.779 | USP28 |
|  | 219221_at | 0.2451 | 0.664 | ZBTB38 |
|  | 205898_at | 0.2471 | 0.679 | CX3CR1 |
|  | 1564139_at | 0.2486 | 0.751 | A2M-AS1 |
|  | 209815_at | 0.2574 | 0.734 | PTCH1 |
|  | 215806_x_at | 0.2582 | 0.811 | NA |
|  | 206991_s_at | 0.3033 | 0.642 | CCR5 |
|  | 242628_at | 0.338 | 0.488 | KLRB1 |
|  | 207723_s_at | 0.3457 | 0.688 | KLRC3 |
|  | 205831_at | 0.357 | 0.719 | CD2 |
|  | 204529_s_at | 0.3696 | 0.783 | TOX |
|  | 226347_at | 0.3835 | 0.795 | FUT11 |
|  | 209993_at | 0.3859 | 0.516 | ABCB1 |
|  | 1553772_at | 0.3871 | 0.675 | GK5 |
|  | 235567_at | 0.4162 | 0.723 | RORA |
|  | 201681_s_at | 0.4291 | 0.653 | DLG5 |
|  | 217629_at | 0.4551 | 0.474 | NA |
|  | 213906_at | 0.4651 | 0.771 | MYBL1 |
|  | 205888_s_at | 0.4753 | 0.552 | JAKMIP2 |
|  | 203216_s_at | 0.4758 | 0.592 | MYO6 |
|  | 224901_at | 0.4766 | 0.333 | SCD5 |
|  | 203989_x_at | 0.4957 | 0.606 | F2R |
|  | 204530_s_at | 0.5139 | 0.489 | TOX |
|  | 231323_at | 0.5143 | 0.468 | PSMB2 |
|  | 1562529_s_at | 0.5344 | 0.627 | NA |
|  | 229689_s_at | 0.5726 | 0.585 | DLG5 |
|  | 225105_at | 0.5784 | 0.660 | C12orf75 |
|  | 227394_at | 0.5982 | 0.499 | NCAM1 |
|  | 226157_at | 0.6803 | 0.654 | TFDP2 |
|  | 218309_at | 0.701 | 0.515 | CAMK2N1 |
|  | 221092_at | 0.7452 | 0.767 | IKZF3 |
|  | 235171_at | 0.7823 | 0.682 | LOC100505501 |
|  | 1564160_at | 0.8163 | 0.665 | DTHD1 |
|  | 215388_s_at | 0.8359 | 0.504 | NA |
|  | 207840_at | 0.8782 | 0.552 | CD160 |
|  | 227188_at | 0.9071 | 0.550 | EVA1C |
|  | 1566442_at | 0.9447 | 0.488 | NA |
|  | 228256_s_at | 0.9808 | 0.590 | EPB41L4A |

Table S8. Lightgreen module contents and information. Hub gene is highlighted.

| **Module information** | **Probe** | **GS (p-value)** | **MM (module cor.)** | **Gene symbol** |
| --- | --- | --- | --- | --- |
| Module name: lightgreen  Number of probes: 147  Minimum self correlation: 0.46  Mean self correlation: 0.65 | 208268_at | 0.0003565 | 0.593 | ADAM28 |
|  | 1566880_at | 0.000669 | 0.637 | NA |
|  | 222915_s_at | 0.0008998 | 0.705 | BANK1 |
|  | 227198_at | 0.001406 | 0.800 | AFF3 |
|  | 219073_s_at | 0.00172 | 0.767 | OSBPL10 |
|  | 228599_at | 0.001861 | 0.850 | MS4A1 |
|  | 235401_s_at | 0.003573 | 0.650 | FCRLA |
|  | 230245_s_at | 0.003582 | 0.721 | LINC00926 |
|  | 232112_at | 0.003785 | 0.749 | RALGPS2 |
|  | 243968_x_at | 0.007092 | 0.892 | FCRL1 |
|  | 203642_s_at | 0.007263 | 0.635 | COBLL1 |
|  | 235372_at | 0.007428 | 0.676 | FCRLA |
|  | 210356_x_at | 0.007442 | 0.817 | MS4A1 |
|  | 1559078_at | 0.009301 | 0.738 | NA |
|  | 224405_at | 0.01016 | 0.663 | FCRL5 |
|  | 219497_s_at | 0.01268 | 0.808 | BCL11A |
|  | 231418_at | 0.01288 | 0.833 | MS4A1 |
|  | 233261_at | 0.01349 | 0.830 | EBF1 |
|  | 235982_at | 0.01456 | 0.607 | FCRL1 |
|  | 237625_s_at | 0.01506 | 0.771 | IGKC |
|  | 1558662_s_at | 0.01581 | 0.885 | BANK1 |
|  | 225081_s_at | 0.01695 | 0.492 | CDCA7L |
|  | 241446_at | 0.01747 | 0.766 | ADAM28 |
|  | 233251_at | 0.0182 | 0.801 | STRBP |
|  | 227224_at | 0.01935 | 0.811 | RALGPS2 |
|  | 236414_at | 0.02043 | 0.762 | NA |
|  | 1563876_at | 0.02101 | 0.580 | C1orf220 |
|  | 229487_at | 0.02149 | 0.557 | EBF1 |
|  | 219667_s_at | 0.02166 | 0.895 | BANK1 |
|  | 205544_s_at | 0.02192 | 0.518 | CR2 |
|  | 217418_x_at | 0.02199 | 0.809 | MS4A1 |
|  | 229513_at | 0.02336 | 0.879 | STRBP |
|  | 227533_at | 0.02401 | 0.784 | NA |
|  | 224339_s_at | 0.02479 | 0.735 | ANGPTL1 |
|  | 1554343_a_at | 0.02506 | 0.768 | STAP1 |
|  | 212387_at | 0.0258 | 0.697 | TCF4 |
|  | 1563674_at | 0.03248 | 0.821 | FCRL2 |
|  | 209307_at | 0.03527 | 0.706 | SWAP70 |
|  | 242458_at | 0.03793 | 0.861 | RALGPS2 |
|  | 239619_at | 0.03841 | 0.573 | NA |
|  | 228003_at | 0.03886 | 0.778 | RAB30 |
|  | 226150_at | 0.03936 | 0.756 | PPAPDC1B |
|  | 223245_at | 0.0452 | 0.789 | STRBP |
|  | 231647_s_at | 0.04776 | 0.678 | FCRL5 |
|  | 228818_at | 0.05007 | 0.707 | NA |
|  | 207655_s_at | 0.05273 | 0.862 | BLNK |
|  | 239183_at | 0.05289 | 0.762 | ANGPTL1 |
|  | 203753_at | 0.06282 | 0.584 | TCF4 |
|  | 1554636_at | 0.06584 | 0.582 | NA |
|  | 213600_at | 0.06837 | 0.628 | SIPA1L3 |
|  | 205997_at | 0.06881 | 0.589 | ADAM28 |
|  | 1563553_at | 0.06921 | 0.609 | NA |
|  | 205267_at | 0.0707 | 0.671 | POU2AF1 |
|  | 228390_at | 0.07194 | 0.717 | RAB30 |
|  | 243867_at | 0.07282 | 0.669 | NA |
|  | 219498_s_at | 0.07512 | 0.842 | BCL11A |
|  | 221586_s_at | 0.0754 | 0.754 | E2F5 |
|  | 1554306_at | 0.07656 | 0.515 | ITPKB |
|  | 233252_s_at | 0.07985 | 0.844 | STRBP |
|  | 228592_at | 0.08292 | 0.888 | MS4A1 |
|  | 226694_at | 0.08609 | 0.657 | NA |
|  | 1562453_at | 0.09483 | 0.571 | NA |
|  | 229072_at | 0.1044 | 0.636 | RAB30 |
|  | 205801_s_at | 0.1119 | 0.613 | RASGRP3 |
|  | 1558186_s_at | 0.1169 | 0.754 | NA |
|  | 1563189_at | 0.1276 | 0.699 | NA |
|  | 204005_s_at | 0.1401 | 0.714 | PAWR |
|  | 1558185_at | 0.1408 | 0.597 | NA |
|  | 214806_at | 0.1503 | 0.626 | BICD1 |
|  | 218149_s_at | 0.1518 | 0.580 | ZNF395 |
|  | 233951_at | 0.1534 | 0.642 | NA |
|  | 209306_s_at | 0.1619 | 0.819 | SWAP70 |
|  | 217902_s_at | 0.1627 | 0.504 | HERC2 |
|  | 240593_x_at | 0.1693 | 0.610 | NA |
|  | 1555890_at | 0.1712 | 0.543 | NA |
|  | 231329_at | 0.1722 | 0.719 | NA |
|  | 231500_s_at | 0.1727 | 0.524 | BOLA2 |
|  | 234428_at | 0.1884 | 0.682 | NA |
|  | 208118_x_at | 0.1884 | 0.597 | NA |
|  | 220059_at | 0.1905 | 0.775 | STAP1 |
|  | 242580_at | 0.2053 | 0.506 | NA |
|  | 227646_at | 0.2196 | 0.661 | EBF1 |
|  | 214602_at | 0.2276 | 0.669 | COL4A4 |
|  | 216753_at | 0.2284 | 0.585 | NA |
|  | 231002_s_at | 0.2315 | 0.531 | RABEP1 |
|  | 216574_s_at | 0.2329 | 0.543 | NA |
|  | 224392_s_at | 0.2375 | 0.502 | OPN3 |
|  | 202759_s_at | 0.2452 | 0.604 | NA |
|  | 212382_at | 0.2508 | 0.829 | TCF4 |
|  | 226713_at | 0.2566 | 0.658 | CCDC50 |
|  | 201691_s_at | 0.2593 | 0.770 | TPD52 |
|  | 230865_at | 0.2668 | 0.693 | LIX1 |
|  | 237907_at | 0.2698 | 0.561 | NA |
|  | 206983_at | 0.2777 | 0.681 | CCR6 |
|  | 227842_at | 0.278 | 0.732 | RAB30 |
|  | 235987_at | 0.2926 | 0.472 | PRKXP1 |
|  | 222891_s_at | 0.2993 | 0.759 | BCL11A |
|  | 239244_at | 0.2995 | 0.644 | LOC100507616 |
|  | 241844_x_at | 0.3099 | 0.530 | TMEM156 |
|  | 239265_at | 0.3105 | 0.469 | SLC35G1 |
|  | 213891_s_at | 0.3162 | 0.724 | TCF4 |
|  | 230232_at | 0.3401 | 0.681 | SEL1L3 |
|  | 234164_at | 0.3669 | 0.658 | NA |
|  | 222051_s_at | 0.3834 | 0.639 | E2F5 |
|  | 223714_at | 0.3946 | 0.520 | ZNF256 |
|  | 212386_at | 0.412 | 0.724 | TCF4 |
|  | 202760_s_at | 0.4188 | 0.817 | NA |
|  | 228711_at | 0.4216 | 0.514 | ZNF37A |
|  | 238828_at | 0.4314 | 0.540 | KIAA1919 |
|  | 223766_at | 0.4464 | 0.599 | LOC100133130 |
|  | 239016_at | 0.4506 | 0.468 | NA |
|  | 1553645_at | 0.4656 | 0.611 | CCDC141 |
|  | 241834_at | 0.4778 | 0.617 | NA |
|  | 231773_at | 0.4833 | 0.692 | ANGPTL1 |
|  | 229126_at | 0.5025 | 0.481 | TMEM19 |
|  | 226203_at | 0.5026 | 0.523 | MYO9A |
|  | 1559186_at | 0.5277 | 0.506 | PRKXP1 |
|  | 230618_s_at | 0.5393 | 0.618 | PRRC2C |
|  | 210893_at | 0.5593 | 0.630 | NA |
|  | 223246_s_at | 0.5635 | 0.657 | STRBP |
|  | 205340_at | 0.5668 | 0.533 | ZBTB24 |
|  | 221768_at | 0.5675 | 0.619 | SFPQ |
|  | 235813_at | 0.5734 | 0.494 | NA |
|  | 244609_at | 0.5909 | 0.566 | NA |
|  | 214041_x_at | 0.6011 | 0.487 | RPL37A |
|  | 230454_at | 0.6518 | 0.519 | ICA1L |
|  | 1566108_at | 0.685 | 0.569 | MYNN |
|  | 227979_at | 0.69 | 0.575 | RBM4 |
|  | 238691_at | 0.7129 | 0.465 | SNHG10 |
|  | 1559188_x_at | 0.7166 | 0.481 | PRKXP1 |
|  | 1562412_at | 0.7169 | 0.559 | NA |
|  | 235353_at | 0.7407 | 0.569 | SEL1L3 |
|  | 207362_at | 0.7547 | 0.559 | SLC30A4 |
|  | 241930_x_at | 0.7824 | 0.516 | NA |
|  | 239014_at | 0.8014 | 0.644 | CCAR1 |
|  | 235526_at | 0.8309 | 0.523 | NA |
|  | 235603_at | 0.8353 | 0.535 | HNRNPU |
|  | 1556175_at | 0.8485 | 0.614 | MTSS1L |
|  | 235012_at | 0.859 | 0.531 | LRCH1 |
|  | 201103_x_at | 0.8606 | 0.505 | NA |
|  | 220567_at | 0.8735 | 0.592 | IKZF2 |
|  | 237963_x_at | 0.9128 | 0.515 | NA |
|  | 240062_at | 0.9428 | 0.602 | FAM3C |
|  | 223712_at | 0.9477 | 0.473 | PCBD2 |
|  | 234132_at | 0.9695 | 0.459 | NA |
|  | 244791_at | 0.9882 | 0.561 | NA |
|  | 1555388_s_at | 0.9973 | 0.548 | SNX25 |

Table S9. Darkturquoise module contents and information. Hub gene is highlighted.

| **Module information** | **Probe** | **GS (p-value)** | **MM (module cor.)** | **Gene symbol** |
| --- | --- | --- | --- | --- |
| Module name: darkturquoise  Number of probes: 73  Minimum self correlation: 0.46  Mean self correlation: 0.67 | 212070_at | 0.0002425 | 0.804 | GPR56 |
|  | 232223_at | 0.002294 | 0.775 | NA |
|  | 242504_at | 0.003791 | 0.517 | NA |
|  | 214617_at | 0.006696 | 0.819 | PRF1 |
|  | 215345_x_at | 0.007809 | 0.687 | TARP |
|  | 211333_s_at | 0.01213 | 0.594 | FASLG |
|  | 219159_s_at | 0.01421 | 0.735 | SLAMF7 |
|  | 237846_at | 0.01441 | 0.667 | NA |
|  | 240188_at | 0.01623 | 0.601 | NA |
|  | 238600_at | 0.01645 | 0.546 | JAKMIP1 |
|  | 215332_s_at | 0.0308 | 0.627 | CD8B |
|  | 234306_s_at | 0.04118 | 0.768 | SLAMF7 |
|  | 213915_at | 0.04674 | 0.904 | NKG7 |
|  | 210031_at | 0.05232 | 0.704 | CD247 |
|  | 1558733_at | 0.05392 | 0.657 | ZBTB38 |
|  | 230464_at | 0.05665 | 0.871 | S1PR5 |
|  | 235110_at | 0.05808 | 0.621 | PLA2G16 |
|  | 204103_at | 0.05932 | 0.760 | CCL4 |
|  | 210164_at | 0.06847 | 0.764 | GZMB |
|  | 220684_at | 0.06969 | 0.834 | TBX21 |
|  | 205291_at | 0.0706 | 0.643 | IL2RB |
|  | 211685_s_at | 0.07503 | 0.649 | NCALD |
|  | 206099_at | 0.07514 | 0.796 | PRKCH |
|  | 1553681_a_at | 0.08007 | 0.835 | PRF1 |
|  | 217374_x_at | 0.08603 | 0.702 | NA |
|  | 217381_s_at | 0.08894 | 0.696 | TARP |
|  | 207351_s_at | 0.09612 | 0.725 | SH2D2A |
|  | 242873_at | 0.1005 | 0.567 | NA |
|  | 1559916_a_at | 0.1015 | 0.716 | NA |
|  | 1555486_a_at | 0.105 | 0.710 | PRR5L |
|  | 226549_at | 0.1168 | 0.712 | SBK1 |
|  | 1566608_at | 0.1223 | 0.702 | NA |
|  | 1555759_a_at | 0.1344 | 0.745 | CCL5 |
|  | 230037_at | 0.141 | 0.594 | CD8B |
|  | 206746_at | 0.1411 | 0.558 | BFSP1 |
|  | 205495_s_at | 0.1444 | 0.794 | GNLY |
|  | 234186_at | 0.1527 | 0.599 | NA |
|  | 214083_at | 0.1527 | 0.502 | PPP2R5C |
|  | 201565_s_at | 0.1624 | 0.682 | ID2 |
|  | 37145_at | 0.1802 | 0.781 | GNLY |
|  | 228658_at | 0.1934 | 0.717 | MIAT |
|  | 213305_s_at | 0.2047 | 0.686 | PPP2R5C |
|  | 237322_at | 0.2228 | 0.708 | MIAT |
|  | 1568934_at | 0.2393 | 0.742 | CX3CR1 |
|  | 240607_at | 0.2499 | 0.592 | MIAT |
|  | 204655_at | 0.2502 | 0.813 | CCL5 |
|  | 242359_at | 0.2509 | 0.563 | NA |
|  | 1563649_at | 0.2525 | 0.670 | NA |
|  | 212566_at | 0.2746 | 0.598 | MAP4 |
|  | 236557_at | 0.2773 | 0.610 | ZBTB38 |
|  | 220120_s_at | 0.2962 | 0.520 | EPB41L4A |
|  | 227168_at | 0.3339 | 0.618 | MIAT |
|  | 1562698_x_at | 0.345 | 0.704 | LOC339988 |
|  | 218927_s_at | 0.3604 | 0.604 | CHST12 |
|  | 237014_at | 0.3609 | 0.704 | MYBL1 |
|  | 1562697_at | 0.3704 | 0.686 | LOC339988 |
|  | 204197_s_at | 0.3856 | 0.842 | RUNX3 |
|  | 1405_i_at | 0.4282 | 0.782 | CCL5 |
|  | 204346_s_at | 0.4635 | 0.613 | RASSF1 |
|  | 214567_s_at | 0.4822 | 0.540 | NA |
|  | 233708_at | 0.5171 | 0.666 | NA |
|  | 223413_s_at | 0.5338 | 0.568 | LYAR |
|  | 206366_x_at | 0.5685 | 0.527 | XCL1 |
|  | 1552497_a_at | 0.6676 | 0.782 | SLAMF6 |
|  | 216748_at | 0.6833 | 0.610 | PYHIN1 |
|  | 227819_at | 0.7456 | 0.636 | LGR6 |
|  | 207979_s_at | 0.7929 | 0.538 | CD8B |
|  | 223126_s_at | 0.8261 | 0.545 | C1orf21 |
|  | 220307_at | 0.827 | 0.674 | CD244 |
|  | 235651_at | 0.8836 | 0.666 | TTC22 |
|  | 212729_at | 0.8863 | 0.604 | DLG3 |
|  | 240070_at | 0.9668 | 0.464 | TIGIT |
|  | 232686_at | 0.9739 | 0.574 | SIGLEC17P |

Table S10. Darkgrey module contents and information. Hub genes are highlighted.

| **Module information** | **Probe** | **GS (p-value)** | **MM (module cor.)** | **Gene symbol** |
| --- | --- | --- | --- | --- |
| Module name: darkgrey  Number of probes: 65  Minimum self correlation: 0.65  Mean self correlation: 0.81 | 229625_at | 0.00856 | 0.767 | GBP5 |
|  | 238581_at | 0.01589 | 0.769 | GBP5 |
|  | 239979_at | 0.02265 | 0.806 | NA |
|  | 202269_x_at | 0.02799 | 0.871 | GBP1 |
|  | 224225_s_at | 0.03317 | 0.741 | ETV7 |
|  | 219684_at | 0.03471 | 0.780 | RTP4 |
|  | 1561738_at | 0.03523 | 0.746 | NA |
|  | 237622_at | 0.03614 | 0.843 | NA |
|  | 206133_at | 0.0476 | 0.918 | XAF1 |
|  | 219716_at | 0.0502 | 0.768 | APOL6 |
|  | 202748_at | 0.05144 | 0.688 | GBP2 |
|  | 208965_s_at | 0.05365 | 0.822 | IFI16 |
|  | 214453_s_at | 0.05403 | 0.872 | IFI44 |
|  | 235574_at | 0.05935 | 0.721 | GBP4 |
|  | 223834_at | 0.06348 | 0.678 | CD274 |
|  | 225931_s_at | 0.06771 | 0.806 | RNF213 |
|  | 231577_s_at | 0.06873 | 0.867 | GBP1 |
|  | 232375_at | 0.08769 | 0.698 | NA |
|  | 214059_at | 0.0889 | 0.854 | IFI44 |
|  | 226603_at | 0.09018 | 0.859 | SAMD9L |
|  | 201649_at | 0.09329 | 0.827 | UBE2L6 |
|  | 227609_at | 0.09872 | 0.900 | EPSTI1 |
|  | 237538_at | 0.1001 | 0.808 | NA |
|  | 1557116_at | 0.1077 | 0.745 | APOL6 |
|  | 219352_at | 0.1141 | 0.731 | HERC6 |
|  | 224701_at | 0.1243 | 0.901 | PARP14 |
|  | 219863_at | 0.1291 | 0.895 | HERC5 |
|  | 233425_at | 0.1332 | 0.706 | NA |
|  | 243271_at | 0.1379 | 0.904 | NA |
|  | 232610_at | 0.146 | 0.820 | PARP14 |
|  | AFFX-HUMISGF3A/M97935_MA_at | 0.1574 | 0.757 | STAT1 |
|  | 235175_at | 0.1592 | 0.687 | GBP4 |
|  | AFFX-HUMISGF3A/M97935_MB_at | 0.1692 | 0.847 | STAT1 |
|  | 238743_at | 0.1767 | 0.745 | NA |
|  | 227807_at | 0.1834 | 0.759 | PARP9 |
|  | 213797_at | 0.185 | 0.892 | RSAD2 |
|  | 203157_s_at | 0.1952 | 0.719 | GLS |
|  | AFFX-HUMISGF3A/M97935_5_at | 0.1957 | 0.828 | STAT1 |
|  | 225636_at | 0.2075 | 0.798 | STAT2 |
|  | 235276_at | 0.2119 | 0.800 | EPSTI1 |
|  | 217502_at | 0.2584 | 0.888 | IFIT2 |
|  | 228617_at | 0.26 | 0.890 | XAF1 |
|  | 242898_at | 0.2605 | 0.834 | EIF2AK2 |
|  | 242625_at | 0.2746 | 0.883 | RSAD2 |
|  | 203153_at | 0.2901 | 0.872 | IFIT1 |
|  | 204415_at | 0.291 | 0.793 | IFI6 |
|  | 244796_at | 0.3157 | 0.791 | NA |
|  | 209969_s_at | 0.3219 | 0.838 | STAT1 |
|  | 209762_x_at | 0.3275 | 0.737 | SP110 |
|  | 230036_at | 0.3362 | 0.856 | SAMD9L |
|  | 202086_at | 0.3422 | 0.782 | MX1 |
|  | 1556297_a_at | 0.3462 | 0.813 | NA |
|  | 218400_at | 0.3481 | 0.878 | OAS3 |
|  | 222816_s_at | 0.365 | 0.855 | ZCCHC2 |
|  | 204439_at | 0.3794 | 0.876 | IFI44L |
|  | 222793_at | 0.4031 | 0.847 | DDX58 |
|  | 201786_s_at | 0.4056 | 0.712 | ADAR |
|  | 236156_at | 0.4272 | 0.875 | LIPA |
|  | 204747_at | 0.446 | 0.801 | IFIT3 |
|  | 229450_at | 0.4538 | 0.909 | IFIT3 |
|  | 232464_at | 0.462 | 0.731 | TRIM78P |
|  | 208012_x_at | 0.4777 | 0.738 | SP110 |
|  | 218543_s_at | 0.4907 | 0.785 | PARP12 |
|  | 204211_x_at | 0.5187 | 0.792 | EIF2AK2 |
|  | 220104_at | 0.8174 | 0.649 | ZC3HAV1 |

Table S11. Top pathway analysis results for royalblue module (unique gene symbols n=79).

| **GO category** | **ID** | **Genes in category** | **p-value** | **FDR q-value** |
| --- | --- | --- | --- | --- |
| regulation of cellular macromolecule biosynthetic process | GO:2000112 | 15 | 0.025 | 0.15 |
| transcription, DNA-dependent | GO:0006351 | 15 | 0.028 | 0.15 |
| RNA biosynthetic process | GO:0032774 | 15 | 0.036 | 0.16 |
| regulation of transcription, DNA-dependent | GO:0006355 | 14 | 0.027 | 0.15 |
| regulation of RNA biosynthetic process | GO:2001141 | 14 | 0.029 | 0.15 |
| regulation of transcription from RNA polymerase II promoter | GO:0006357 | 11 | 0.00080 | 0.12 |
| transcription from RNA polymerase II promoter | GO:0006366 | 11 | 0.0023 | 0.12 |
| positive regulation of transcription, DNA-dependent | GO:0045893 | 9 | 0.0022 | 0.12 |
|  |  |  |  |  |
| **KEGG category** | **ID** | **Genes in category** | **p-value** | **FDR q-value** |
| Antigen processing and presentation | 04612 | 4 | 0.00031 | 0.015 |
| Endocytosis | 04144 | 4 | 0.011 | 0.20 |
| Cell adhesion molecules (CAMs) | 04514 | 3 | 0.020 | 0.20 |
| Natural killer cell mediated cytotoxicity | 04650 | 3 | 0.021 | 0.20 |
| Graft-versus-host disease | 5332 | 2 | 0.014 | 0.20 |

Table S12. Top pathway analysis results for lightgreen module (unique gene symbols n=72).

| **GO category** | **ID** | **Genes in category** | **p-value** | **FDR q-value** |
| --- | --- | --- | --- | --- |
| RNA biosynthetic process | GO:0032774 | 18 | 0.0082 | 0.14 |
| regulation of cellular macromolecule biosynthetic process | GO:2000112 | 17 | 0.012 | 0.16 |
| transcription, DNA-dependent | GO:0006351 | 17 | 0.014 | 0.17 |
| regulation of transcription, DNA-dependent | GO:0006355 | 16 | 0.012 | 0.16 |
| regulation of RNA biosynthetic process | GO:2001141 | 16 | 0.013 | 0.17 |
| small GTPase mediated signal transduction | GO:0007264 | 6 | 0.0057 | 0.12 |
| mRNA metabolic process | GO:0016071 | 5 | 0.043 | 0.23 |
| mRNA splicing, via spliceosome | GO:0000398 | 4 | 0.0041 | 0.12 |
|  |  |  |  |  |
| **KEGG category** | **ID** | **Genes in category** | **p-value** | **FDR q-value** |
| B cell receptor signaling pathway | 04662 | 3 | 0.00041 | 0.0097 |
| Hematopoietic cell lineage | 04640 | 2 | 0.013 | 0.16 |

Table S13. Top pathway analysis results for darkturquoise module (unique gene symbols n=46).

| **GO category** | **ID** | **Genes in category** | **p-value** | **FDR q-value** |
| --- | --- | --- | --- | --- |
| regulation of RNA biosynthetic process | GO:2001141 | 10 | 3.8E-002 | 9.3E-002 |
| RNA biosynthetic process | GO:0032774 | 10 | 7.8E-002 | 1.4E-001 |
| regulation of cellular macromolecule biosynthetic process | GO:2000112 | 9 | 1.3E-001 | 1.9E-001 |
| apoptotic process | GO:0006915 | 8 | 6.5E-003 | 4.9E-002 |
| regulation of transcription, DNA-dependent | GO:0006355 | 8 | 1.7E-001 | 2.3E-001 |
| regulation of apoptotic process | GO:0042981 | 6 | 1.7E-002 | 6.2E-002 |
| generation of neurons | GO:0048699 | 6 | 1.7E-002 | 6.2E-002 |
| regulation of T cell activation | GO:0050863 | 4 | 9.0E-004 | 2.4E-002 |
|  |  |  |  |  |
| **KEGG category** | **ID** | **Genes in category** | **p-value** | **FDR q-value** |
| Cytokine-cytokine receptor interaction | 04060 | 6 | 9.4E-005 | 0.0018 |
| Natural killer cell mediated cytotoxicity | 04650 | 5 | 4.2E-005 | 0.0016 |
| Chemokine signaling pathway | 04062 | 4 | 2.2E-003 | 0.012 |
| Allograft rejection | 05330 | 3 | 1.8E-004 | 0.0022 |
| Graft-versus-host disease | 05332 | 3 | 2.4E-004 | 0.0022 |
| Type I diabetes mellitus | 04940 | 3 | 2.8E-004 | 0.0022 |
| Autoimmune thyroid disease | 05320 | 3 | 4.9E-004 | 0.0032 |
| Chagas disease (American trypanosomiasis) | 05142 | 3 | 3.6E-003 | 0.018 |

Table S14. Top pathway analysis results for darkgrey module (unique gene symbols n=39).

| **GO category** | **ID** | **Genes in category** | **p-value** | **FDR q-value** |
| --- | --- | --- | --- | --- |
| cellular response to cytokine stimulus | GO:0071345 | 15 | 8.5E-016 | 6.9E-014 |
| cytokine-mediated signaling pathway | GO:0019221 | 14 | 8.8E-016 | 6.9E-014 |
| type I interferon-mediated signaling pathway | GO:0060337 | 11 | 1.3E-018 | 2.0E-016 |
| cellular response to type I interferon | GO:0071357 | 11 | 1.3E-018 | 2.0E-016 |
| apoptotic process | GO:0006915 | 8 | 1.0E-002 | 5.9E-002 |
| cellular response to interferon-gamma | GO:0071346 | 5 | 1.3E-006 | 5.7E-005 |
| regulation of apoptotic process | GO:0042981 | 5 | 7.3E-002 | 1.8E-001 |
| response to interferon-alpha | GO:0035455 | 4 | 2.4E-008 | 1.5E-006 |
|  |  |  |  |  |
| **KEGG category** | **ID** | **Genes in category** | **p-value** | **FDR q-value** |
| Hepatitis C | 05160 | 6 | 1.0E-007 | 2.5E-006 |
| Cytosolic DNA-sensing pathway | 04623 | 2 | 5.5E-003 | 6.5E-002 |
| Osteoclast differentiation | 04380 | 2 | 2.7E-002 | 1.3E-001 |
| Jak-STAT signaling pathway | 04630 | 2 | 3.8E-002 | 1.3E-001 |
| Chemokine signaling pathway | 04062 | 2 | 5.5E-002 | 1.3E-001 |
| D-Glutamine and D-glutamate metabolism | 00471 | 1 | 8.2E-003 | 6.5E-002 |
| Steroid biosynthesis | 00100 | 1 | 3.8E-002 | 1.3E-001 |
| Nitrogen metabolism | 00910 | 1 | 4.6E-002 | 1.3E-001 |


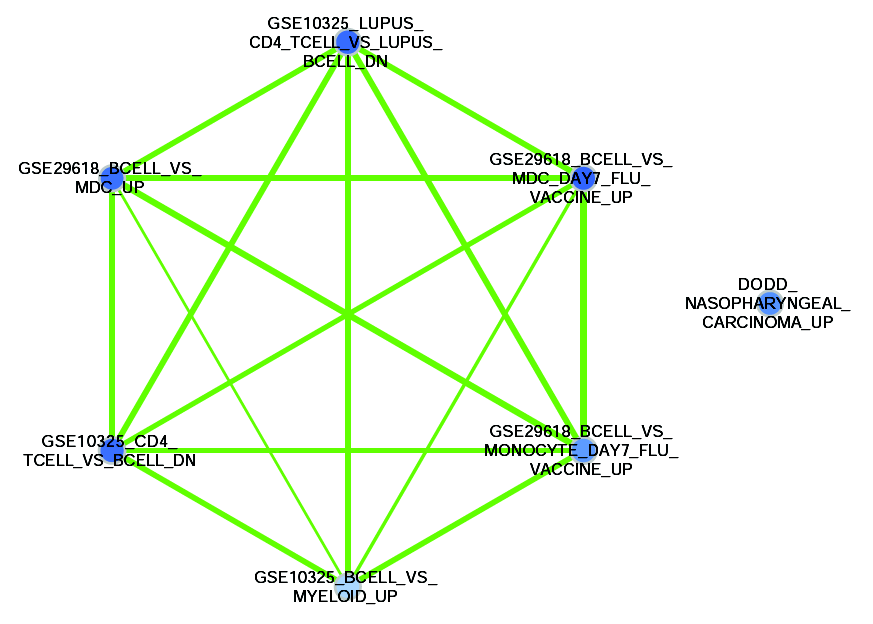


Figure S3. EnrichmentMap network for GSEA results from lightgreen module. Nodes correspond to gene sets and edges correspond to an overlap of the genes within two sets.

Table S15. GSEA results for probe association with exacerbations phenotype

| **correlation** | **Gene set description** | **ID** | **Set overlap** | **P-value** | **FDR q-value** |
| --- | --- | --- | --- | --- | --- |
| **positive** | NAIVE_VS_PD1LOW_CD8_TCELL_DN | GSE26495 | 31 | 0.000 | 0.000 |
|  | NAIVE_VS_PD1HIGH_CD8_TCELL_DN | GSE26495 | 27 | 0.000 | 0.000 |
|  | UNSTIM_VS_NEWCATSLE_VIRUS_DC_6H_DN | GSE18791 | 19 | 0.000 | 0.000 |
|  | UNSTIM_VS_NEWCATSLE_VIRUS_DC_10H_DN | GSE18791 | 19 | 0.000 | 0.000 |
|  | UNSTIM_VS_4H_LPS_DC_TRANSLATED_RNA_DN | GSE14000 | 15 | 0.000 | 0.000 |
|  | HEALTHY_VS_RSV_INF_INFANT_PBMC_UP | GSE34205 | 19 | 0.000 | 0.000 |
|  | DEURIG_T_CELL_PROLYMPHOCYTIC_LEUKEMIA_DN |  | 26 | 0.000 | 0.000 |
|  | CTRL_VS_NEWCASTLE_VIRUS_DC_8H_DN | GSE18791 | 15 | 0.000 | 0.000 |
|  | CENT_MEMORY_CD4_TCELL_VS_NKCELL_DN | GSE3982 | 20 | 0.000 | 0.000 |
|  | CTRL_VS_DAY3_YF17D_VACCINE_PBMC_DN | GSE13485 | 16 | 0.000 | 0.001 |
|  | BOSCO_TH1_CYTOTOXIC_MODULE |  | 16 | 0.000 | 0.001 |
|  | CD4_TCELL_VS_LUPUS_CD4_TCELL_DN | GSE10325 | 19 | 0.000 | 0.001 |
|  | UNSTIM_VS_4H_LPS_DC_DN | GSE14000 | 17 | 0.000 | 0.001 |
|  | NKCELL_VS_SPLENOCYTE_UP | GSE7764 | 22 | 0.000 | 0.002 |
|  | CTRL_VS_IFNG_24H_MICROGLIA_DN | GSE1432 | 19 | 0.000 | 0.005 |
|  | 2H_VS_8H_R848_STIM_DC_DN | GSE2706 | 16 | 0.000 | 0.007 |
|  | CTRL_VS_NEWCASTLE_VIRUS_DC_16H_DN | GSE18791 | 18 | 0.000 | 0.017 |
|  | SENGUPTA_NASOPHARYNGEAL_CARCINOMA_UP |  | 19 | 0.000 | 0.021 |
|  | CTRL_VS_ACT_IL4_AND_ANTI_IL12_72H_CD4_TCELL_DN | GSE17974 | 15 | 0.000 | 0.028 |
|  | UNSTIM_VS_YF17D_VACCINE_STIM_PBMC_DN | GSE13484 | 17 | 0.000 | 0.031 |
|  | 1H_VS_24H_IFNG_MICROGLIA_DN | GSE1432 | 15 | 0.000 | 0.032 |
|  | DODD_NASOPHARYNGEAL_CARCINOMA_DN |  | 74 | 0.003 | 0.038 |
|  | NAIVE_CD8_TCELL_VS_MEMORY_TCELL_UP | GSE22886 | 25 | 0.002 | 0.037 |
|  | CONTROLLER_VS_PROGRESSOR_HIV_SPECIFIC_CD8_TCELL_DN | GSE24081 | 18 | 0.005 | 0.047 |
|  | CHARAFE_BREAST_CANCER_LUMINAL_VS_BASAL_DN |  | 25 | 0.002 | 0.049 |
|  | RECEPTOR_ACTIVITY |  | 26 | 0.002 | 0.051 |
|  | BILD_HRAS_ONCOGENIC_SIGNATURE |  | 17 | 0.005 | 0.053 |
|  | CTRL_VS_IFNG_6H_MICROGLIA_DN | GSE1432 | 16 | 0.005 | 0.054 |
|  | TREG_VS_NAIVE_CD4_TCELL_DN | GSE20366 | 16 | 0.002 | 0.054 |
|  | TRANSMEMBRANE_RECEPTOR_ACTIVITY |  | 22 | 0.005 | 0.057 |
|  | CELL_CELL_SIGNALING |  | 15 | 0.007 | 0.058 |
|  | TAKEDA_TARGETS_OF_NUP98_HOXA9_FUSION_10D_UP |  | 17 | 0.011 | 0.087 |
|  | CD8_TCELL_VS_CD4_TCELL_ACT_UP | GSE7460 | 15 | 0.009 | 0.088 |
|  | GOZGIT_ESR1_TARGETS_DN |  | 57 | 0.010 | 0.089 |
|  | UNSTIM_VS_8H_LPS_DC_DN | GSE2706 | 18 | 0.010 | 0.087 |
|  | RODWELL_AGING_KIDNEY_NO_BLOOD_UP |  | 15 | 0.009 | 0.089 |
|  | DAY6_VS_DAY10_EFF_CD8_TCELL_UP | GSE15750 | 15 | 0.004 | 0.088 |
|  | HEALTHY_VS_STREP_PNEUMO_INF_PBMC_UP | GSE6269 | 15 | 0.014 | 0.090 |
|  | 12H_VS_3H_YF17D_VACCINE_STIM_PBMC_DN | GSE13484 | 15 | 0.014 | 0.094 |
|  | NAIVE_CD8_TCELL_VS_MONOCYTE_UP | GSE22886 | 22 | 0.017 | 0.092 |
|  | WALLACE_PROSTATE_CANCER_RACE_UP |  | 26 | 0.002 | 0.101 |
|  | V$ISRE_01 |  | 21 | 0.007 | 0.107 |
|  | MATSUDA_NATURAL_KILLER_DIFFERENTIATION |  | 42 | 0.007 | 0.105 |
|  | NUYTTEN_NIPP1_TARGETS_UP |  | 36 | 0.005 | 0.105 |
|  | CYTOPLASMIC_PART |  | 61 | 0.005 | 0.108 |
|  | SENESE_HDAC1_TARGETS_UP |  | 24 | 0.009 | 0.122 |
|  | JAATINEN_HEMATOPOIETIC_STEM_CELL_UP |  | 24 | 0.009 | 0.124 |
|  | THYMUS_VS_FAT_TREG_DN | GSE7852 | 15 | 0.027 | 0.132 |
|  | 2H_VS_8H_R848_AND_LPS_STIM_DC_DN | GSE2706 | 19 | 0.021 | 0.134 |
|  | KEGG_MAPK_SIGNALING_PATHWAY |  | 15 | 0.013 | 0.146 |
|  | PD1_LIGATION_VS_CTRL_IN_ACT_TCELL_LINE_UP | GSE24026 | 15 | 0.027 | 0.147 |
|  | TAKEDA_TARGETS_OF_NUP98_HOXA9_FUSION_16D_UP |  | 17 | 0.019 | 0.157 |
|  | BASAKI_YBX1_TARGETS_DN |  | 28 | 0.021 | 0.157 |
|  | CHICAS_RB1_TARGETS_CONFLUENT |  | 38 | 0.014 | 0.172 |
|  | STREP_AUREUS_VS_STREP_PNEUMO_INF_PBMC_UP | GSE6269 | 15 | 0.018 | 0.174 |
|  | UNSTIM_VS_8H_LPS_AND_R848_DC_DN | GSE2706 | 15 | 0.033 | 0.179 |
|  | PASINI_SUZ12_TARGETS_DN |  | 16 | 0.022 | 0.181 |
|  | CD2_POS_VS_NEG_PDC_UP | GSE15215 | 15 | 0.019 | 0.179 |
|  | SCHUETZ_BREAST_CANCER_DUCTAL_INVASIVE_UP |  | 16 | 0.018 | 0.177 |
|  | LI_INDUCED_T_TO_NATURAL_KILLER_UP |  | 17 | 0.023 | 0.177 |
|  | NUYTTEN_EZH2_TARGETS_UP |  | 61 | 0.013 | 0.177 |
|  | CD8_TCELL_VS_BCELL_NAIVE_UP | GSE22886 | 17 | 0.022 | 0.179 |
|  | PD1HIGH_VS_PD1LOW_CD8_TCELL_DN | GSE26495 | 15 | 0.032 | 0.189 |
|  | LEE_BMP2_TARGETS_UP |  | 28 | 0.014 | 0.189 |
|  | HELLER_SILENCED_BY_METHYLATION_UP |  | 19 | 0.026 | 0.203 |
|  | KEGG_PATHWAYS_IN_CANCER |  | 20 | 0.025 | 0.205 |
|  | TRANSPORT |  | 44 | 0.030 | 0.248 |
|  |  |  |  |  |  |
| **negative** | CD4_TCELL_VS_BCELL_DN | GSE10325 | 48 | 0.000 | 0.000 |
|  | LUPUS_CD4_TCELL_VS_LUPUS_BCELL_DN | GSE10325 | 45 | 0.000 | 0.000 |
|  | BCELL_VS_MONOCYTE_UP | GSE29618 | 43 | 0.000 | 0.000 |
|  | BCELL_VS_MONOCYTE_DAY7_FLU_VACCINE_UP | GSE29618 | 41 | 0.000 | 0.000 |
|  | BCELL_VS_MYELOID_UP | GSE10325 | 34 | 0.000 | 0.000 |
|  | BCELL_VS_MDC_DAY7_FLU_VACCINE_UP | GSE29618 | 35 | 0.000 | 0.000 |
|  | NAIVE_BCELL_VS_NEUTROPHIL_UP | GSE22886 | 28 | 0.000 | 0.000 |
|  | LUPUS_BCELL_VS_LUPUS_MYELOID_UP | GSE10325 | 28 | 0.000 | 0.000 |
|  | BCELL_VS_MDC_UP | GSE29618 | 33 | 0.000 | 0.000 |
|  | MCLACHLAN_DENTAL_CARIES_UP |  | 16 | 0.000 | 0.000 |
|  | HADDAD_B_LYMPHOCYTE_PROGENITOR |  | 34 | 0.000 | 0.000 |
|  | TCELL_VS_BCELL_NAIVE_DN | GSE22886 | 37 | 0.000 | 0.000 |
|  | NAIVE_BCELL_VS_MONOCYTE_UP | GSE22886 | 39 | 0.000 | 0.000 |
|  | BCELL_VS_PDC_DAY7_FLU_VACCINE_UP | GSE29618 | 32 | 0.000 | 0.000 |
|  | BCELL_VS_PDC_UP | GSE29618 | 28 | 0.000 | 0.000 |
|  | BCELL_VS_CENT_MEMORY_CD4_TCELL_UP | GSE3982 | 31 | 0.000 | 0.000 |
|  | MODULE_436 |  | 25 | 0.000 | 0.000 |
|  | CD4_TCELL_VS_BCELL_NAIVE_DN | GSE22886 | 26 | 0.000 | 0.000 |
|  | BCELL_VS_NKCELL_UP | GSE3982 | 30 | 0.000 | 0.000 |
|  | MODULE_345 |  | 22 | 0.000 | 0.000 |
|  | MEMORY_CD4_TCELL_VS_BCELL_DN | GSE3982 | 32 | 0.000 | 0.000 |
|  | LEE_RECENT_THYMIC_EMIGRANT |  | 19 | 0.000 | 0.000 |
|  | NAIVE_VS_MEMORY_BCELL_UP | GSE12366 | 22 | 0.000 | 0.000 |
|  | STREP_AUREUS_VS_STREP_PNEUMO_INF_PBMC_DN | GSE6269 | 21 | 0.000 | 0.000 |
|  | MODULE_292 |  | 25 | 0.000 | 0.000 |
|  | MODULE_188 |  | 22 | 0.000 | 0.000 |
|  | MODULE_238 |  | 21 | 0.000 | 0.001 |
|  | PDC_VS_MDC_DAY7_FLU_VACCINE_UP | GSE29618 | 22 | 0.000 | 0.001 |
|  | MCLACHLAN_DENTAL_CARIES_DN |  | 19 | 0.000 | 0.001 |
|  | MODULE_301 |  | 21 | 0.000 | 0.001 |
|  | CD8_TCELL_VS_BCELL_NAIVE_DN | GSE22886 | 32 | 0.000 | 0.002 |
|  | MODULE_53 |  | 20 | 0.000 | 0.002 |
|  | MONOCYTE_VS_PDC_DAY7_FLU_VACCINE_DN | GSE29618 | 19 | 0.000 | 0.002 |
|  | PBMC_VS_MEM_CD4_TCELL_UP | GSE11057 | 19 | 0.000 | 0.002 |
|  | NAIVE_BCELL_VS_DC_UP | GSE22886 | 27 | 0.000 | 0.002 |
|  | MODULE_208 |  | 19 | 0.000 | 0.003 |
|  | BCELL_VS_EFF_MEMORY_CD4_TCELL_UP | GSE3982 | 21 | 0.000 | 0.003 |
|  | HEALTHY_VS_STREP_AUREUS_INF_PBMC_UP | GSE6269 | 27 | 0.000 | 0.003 |
|  | MODULE_361 |  | 21 | 0.000 | 0.003 |
|  | KAECH_DAY8_EFF_VS_DAY15_EFF_CD8_TCELL_DN |  | 17 | 0.000 | 0.004 |
|  | MODULE_145 |  | 20 | 0.000 | 0.004 |
|  | NAIVE_CD4_VS_PBMC_CD4_TCELL_UP | GSE11057 | 16 | 0.000 | 0.004 |
|  | MODULE_171 |  | 20 | 0.000 | 0.004 |
|  | MONOCYTE_VS_MDC_DN | GSE29618 | 19 | 0.002 | 0.004 |
|  | MONOCYTE_VS_PDC_DN | GSE29618 | 21 | 0.002 | 0.005 |
|  | PASQUALUCCI_LYMPHOMA_BY_GC_STAGE_DN |  | 22 | 0.000 | 0.005 |
|  | PLASMA_CELL_VS_MEMORY_BCELL_DN | GSE13411 | 18 | 0.000 | 0.006 |
|  | KLEIN_PRIMARY_EFFUSION_LYMPHOMA_DN |  | 17 | 0.002 | 0.006 |
|  | KUMAR_TARGETS_OF_MLL_AF9_FUSION |  | 34 | 0.004 | 0.007 |
|  | KAECH_DAY15_EFF_VS_MEMORY_CD8_TCELL_UP |  | 16 | 0.002 | 0.008 |
|  | IGG_IGA_MEMORY_BCELL_VS_BM_PLASMA_CELL_UP | GSE22886 | 16 | 0.002 | 0.009 |
|  | NAIVE_VS_PD1HIGH_CD8_TCELL_UP | GSE26495 | 15 | 0.000 | 0.009 |
|  | DAY3_VS_DAY7_TIV_FLU_VACCINE_PBMC_DN | GSE29614 | 17 | 0.002 | 0.010 |
|  | NAIVE_VS_CENT_MEMORY_CD4_TCELL_UP | GSE11057 | 16 | 0.002 | 0.014 |
|  | NAIVE_TCELL_VS_NKCELL_UP | GSE22886 | 18 | 0.000 | 0.015 |
|  | MONOCYTE_VS_MDC_DAY7_FLU_VACCINE_DN | GSE29618 | 15 | 0.005 | 0.015 |
|  | NAIVE_BCELL_VS_BM_PLASMA_CELL_UP | GSE22886 | 18 | 0.003 | 0.016 |
|  | HEALTHY_VS_FLU_INF_INFANT_PBMC_UP | GSE34205 | 19 | 0.000 | 0.022 |
|  | PRE_GC_VS_DARKZONE_GC_TONSIL_BCELL_UP | GSE12845 | 15 | 0.003 | 0.023 |
|  | VECCHI_GASTRIC_CANCER_EARLY_DN |  | 33 | 0.002 | 0.024 |
|  | DC_VS_BCELL_DN | GSE3982 | 15 | 0.000 | 0.024 |
|  | IGM_MEMORY_BCELL_VS_PLASMA_CELL_UP | GSE13411 | 16 | 0.009 | 0.026 |
|  | MODULE_118 |  | 17 | 0.004 | 0.033 |
|  | 4H_VS_16H_IFNG_IN_CD8POS_DC_DN | GSE3337 | 16 | 0.005 | 0.033 |
|  | BCELL_VS_NKTCELL_UP | GSE27786 | 15 | 0.007 | 0.035 |
|  | SMID_BREAST_CANCER_LUMINAL_B_DN |  | 41 | 0.002 | 0.037 |
|  | NAIVE_VS_IGG_IGA_MEMORY_BCELL_UP | GSE22886 | 17 | 0.005 | 0.041 |
|  | SHEDDEN_LUNG_CANCER_GOOD_SURVIVAL_A12 |  | 27 | 0.005 | 0.044 |
|  | UNINFECTED_VS_S_MANSONI_INF_TREG_UP | GSE17580 | 16 | 0.009 | 0.046 |
|  | CASORELLI_ACUTE_PROMYELOCYTIC_LEUKEMIA_DN |  | 43 | 0.005 | 0.049 |
|  | SMID_BREAST_CANCER_NORMAL_LIKE_UP |  | 55 | 0.000 | 0.054 |
|  | PUJANA_BRCA1_PCC_NETWORK |  | 76 | 0.008 | 0.055 |
|  | NAIVE_VS_PD1LOW_CD8_TCELL_UP | GSE26495 | 19 | 0.007 | 0.054 |
|  | NAIVE_CD4_TCELL_VS_MONOCYTE_UP | GSE22886 | 16 | 0.009 | 0.058 |
|  | NAIVE_TCELL_VS_MONOCYTE_UP | GSE22886 | 15 | 0.004 | 0.058 |
|  | KAECH_NAIVE_VS_MEMORY_CD8_TCELL_UP |  | 18 | 0.011 | 0.065 |
|  | SENGUPTA_NASOPHARYNGEAL_CARCINOMA_DN |  | 26 | 0.012 | 0.066 |
|  | UNINFECTED_VS_S_MANSONI_INF_TEFF_UP | GSE17580 | 19 | 0.016 | 0.067 |
|  | NAIVE_VS_EFF_MEMORY_CD4_TCELL_UP | GSE11057 | 20 | 0.010 | 0.067 |
|  | BERENJENO_TRANSFORMED_BY_RHOA_DN |  | 19 | 0.005 | 0.070 |
|  | SABATES_COLORECTAL_ADENOMA_DN |  | 17 | 0.009 | 0.075 |
|  | LIM_MAMMARY_STEM_CELL_DN |  | 21 | 0.018 | 0.088 |
|  | BCELL_VS_TH1_UP | GSE3982 | 28 | 0.010 | 0.092 |
|  | NAIVE_CD4_TCELL_VS_NEUTROPHIL_UP | GSE22886 | 15 | 0.023 | 0.094 |
|  | NAIVE_BCELL_VS_BLOOD_PLASMA_CELL_UP | GSE22886 | 23 | 0.010 | 0.101 |
|  | OSMAN_BLADDER_CANCER_DN |  | 23 | 0.014 | 0.103 |
|  | NAIVE_VS_MEMORY_CD8_TCELL_UP | GSE9650 | 18 | 0.014 | 0.102 |
|  | PUJANA_ATM_PCC_NETWORK |  | 68 | 0.007 | 0.104 |
|  | POOLA_INVASIVE_BREAST_CANCER_UP |  | 23 | 0.009 | 0.106 |
|  | V$GFI1_01 |  | 17 | 0.022 | 0.106 |
|  | BENPORATH_MYC_MAX_TARGETS |  | 27 | 0.015 | 0.107 |
|  | FARMER_BREAST_CANCER_BASAL_VS_LULMINAL |  | 21 | 0.009 | 0.107 |
|  | PDC_VS_MDC_UP | GSE29618 | 19 | 0.015 | 0.107 |
|  | IGG_IGA_MEMORY_BCELL_VS_BLOOD_PLASMA_CELL_UP | GSE22886 | 19 | 0.020 | 0.108 |
|  | GOLDRATH_NAIVE_VS_MEMORY_CD8_TCELL_UP |  | 15 | 0.020 | 0.118 |
|  | PENG_RAPAMYCIN_RESPONSE_UP |  | 17 | 0.030 | 0.132 |
|  | SENESE_HDAC3_TARGETS_DN |  | 33 | 0.020 | 0.132 |
|  | NKCELL_VS_SPLENOCYTE_DN | GSE7764 | 17 | 0.033 | 0.134 |
|  | REACTOME_GENERIC_TRANSCRIPTION_PATHWAY |  | 18 | 0.032 | 0.140 |
|  | DANG_BOUND_BY_MYC |  | 41 | 0.026 | 0.147 |
|  | DODD_NASOPHARYNGEAL_CARCINOMA_UP |  | 126 | 0.008 | 0.152 |
|  | YAGI_AML_WITH_11Q23_REARRANGED |  | 16 | 0.044 | 0.172 |
|  | DEURIG_T_CELL_PROLYMPHOCYTIC_LEUKEMIA_UP |  | 27 | 0.026 | 0.184 |
|  | LINDGREN_BLADDER_CANCER_CLUSTER_2B |  | 15 | 0.047 | 0.186 |
|  | EFF_MEM_VS_CENT_MEM_CD4_TCELL_DN | GSE11057 | 16 | 0.046 | 0.189 |
|  | NAIVE_BCELL_VS_PLASMA_CELL_UP | GSE13411 | 16 | 0.020 | 0.193 |
|  | ZHOU_INFLAMMATORY_RESPONSE_FIMA_DN |  | 21 | 0.042 | 0.199 |
|  | V$NKX25_02 |  | 21 | 0.036 | 0.220 |
|  | MODULE_15 |  | 18 | 0.046 | 0.227 |
|  | QI_PLASMACYTOMA_UP |  | 19 | 0.044 | 0.228 |
|  | UNSTIM_VS_4H_LPS_DC_UP | GSE14000 | 16 | 0.049 | 0.249 |
|  | MODULE_44 |  | 18 | 0.045 | 0.249 |
