# Supplementary figures and images for: Identifying a gene expression signature of frequent COPD exacerbations in peripheral blood using network methods
Source: BMC Med Genomics. 2015 Jan 13;8:1. doi: 10.1186/s12920-014-0072-y (PMC4302028; doi:10.1186/s12920-014-0072-y)

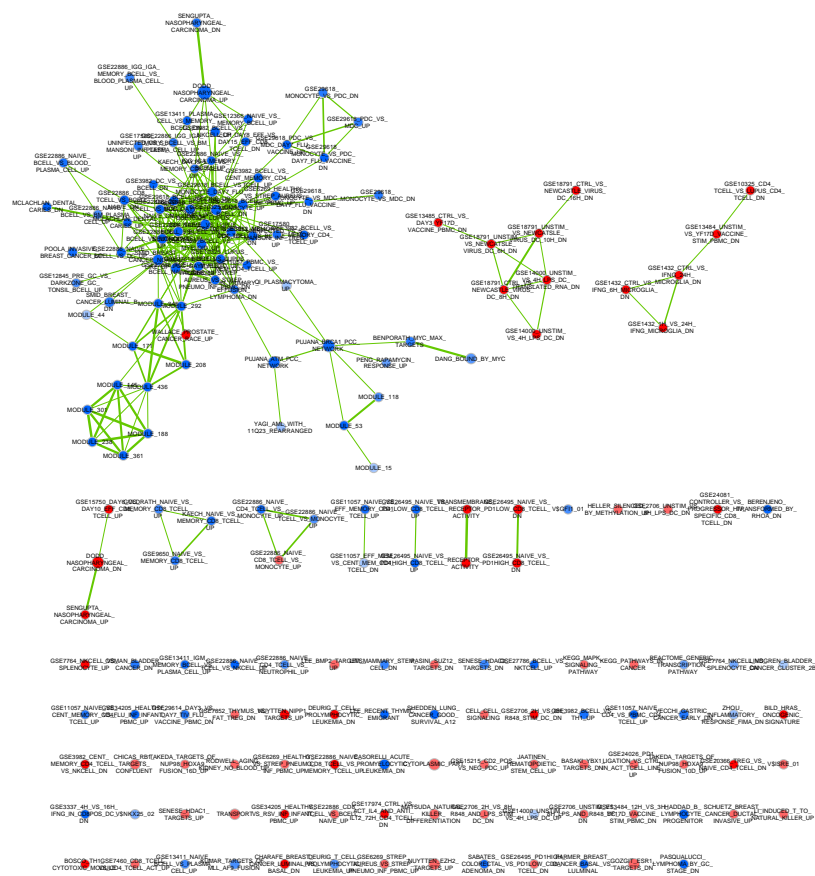

Supplement: Additional file 3: Figure S4. — EnrichmentMap for GSEA results for probe association with exacerbation phenotype. [file 12920_2014_72_MOESM3_ESM.pdf]
